# Systematic assessment of prescribed medications and short-term risk of myocardial infarction – a pharmacopeia-wide association study from Norway and Sweden

**DOI:** 10.1038/s41598-019-44641-1

**Published:** 2019-06-04

**Authors:** Abhijit Sen, Ioannis Vardaxis, Bo Henry Lindqvist, Ben Michael Brumpton, Linn Beate Strand, Inger Johanne Bakken, Lars Johan Vatten, Pål Richard Romundstad, Rickard Ljung, Kenneth Jay Mukamal, Imre Janszky

**Affiliations:** 1 Department of Public health and Nursing, Faculty of Medicine and Health Sciences, Norwegian University of Science and Technology, 7491 Trondheim, Norway; 2 Center for Oral Health Services and Research (TkMidt), Trondheim, Norway; 3 Department of Mathematical Sciences, Norwegian University of Science and Technology, 7491 Trondheim, Norway; 4 Department of Thoracic Medicine, St. Olav’s Hospital, Trondheim University Hospital, Trondheim, Norway; 5 K.G. Jebsen Centre for Genetic Epidemiology, Department of Public Health and Nursing, Norwegian University of Science and Technology, 7491 Trondheim, Norway; 6 MRC Integrative Epidemiology Unit, University of Bristol, Bristol, United Kingdom; 7 Centre for Fertility and Health (CeFH), Norwegian Institute of Public Health, Oslo, Norway; 8 Unit of Epidemiology, Institute of Environmental Medicine, Karolinska Institutet, SE 171 77, Solna, Stockholm Sweden; 9 Department of Medicine, Beth Israel Deaconess Medical Center, Boston, MA United States; 10 Regional Center for Health Care Improvement, St Olav’s Hospital, Trondheim, Norway

## Abstract

Wholesale, unbiased assessment of Scandinavian electronic health-care databases offer a unique opportunity to reveal potentially important undiscovered drug side effects. We examined the short-term risk of acute myocardial infarction (AMI) associated with drugs prescribed in Norway or Sweden. We identified 24,584 and 97,068 AMI patients via the patient- and the cause-of-death registers and linked to prescription databases in Norway (2004–2014) and Sweden (2005–2014), respectively. A case-crossover design was used to compare the drugs dispensed 1–7 days before the date of AMI diagnosis with 15–21 days’ time -window for all the drug individually while controlling the receipt of other drugs. A BOLASSO approach was used to select drugs that acutely either increase or decrease the apparent risk of AMI. We found 48 drugs to be associated with AMI in both countries. Some antithrombotics, antibiotics, opioid analgesics, adrenergics, proton-pump inhibitors, nitroglycerin, diazepam, metoclopramide, acetylcysteine were associated with higher risk for AMI; whereas angiotensin-II-antagonists, calcium-channel blockers, angiotensin-converting-enzyme inhibitors, serotonin-specific reuptake inhibitors, allopurinol, mometasone, metformin, simvastatin, levothyroxine were inversely associated. The results were generally robust in different sensitivity analyses. This study confirms previous findings for certain drugs. Based on the known effects or indications, some other associations could be anticipated. However, inverse associations of hydroxocobalamin, levothyroxine and mometasone were unexpected and needs further investigation. This pharmacopeia-wide association study demonstrates the feasibility of a systematic, unbiased approach to pharmacological triggers of AMI and other diseases with acute, identifiable onsets.

## Introduction

More than 90% of all US adults aged 65 years and older use at least one prescription medication^1^, a proportion that continues to increase. Adverse and often unanticipated drug reactions are a major public health challenge. They are estimated to cost around $30 billion annually in the US alone^2^.

Although drugs undergo several phases of testing prior to approval, pre-approval trials are typically just large enough to detect the expected effect on physiological outcomes. These trials are commonly too small and of too short duration to assess meaningful clinical outcomes^3^. Pre-approval trials also tend to disproportionately exclude women, especially in their reproductive age, patients with comorbidities, individuals in lower socioeconomic strata, children, and elderly individuals, all of which limit generalizability of findings^4^. Thus, an urgent need exists for systematic, generalizable monitoring of important clinical effects even in the post-marketing phase. For example, rofecoxib and sibutramine were both withdrawn from the market due to increased risk for acute myocardial infarction (AMI)^5,6^.

Although many adverse drug reactions can be anticipated on the basis of a drug’s physiological effects, the example of the human genome has illustrated how modest the predictive power of variants in established candidate pathways can be. In the case of genomics, this has been addressed by technological improvements that enable wholesale, unbiased assessment of millions of genetic variants on a single outcome –genome-wide association studies (GWAS). These studies have identified dozens of novel pathways across a variety of endpoints. In the case of pharmaceuticals, the advent of registers that track the dispensing of prescription medications enables a comparable approach – the pharmacopoeia-wide association study (PWAS) – to perform unbiased assessment of literally all prescribed medications. Such an approach could offer the potential to identify drugs that unexpectedly increase or decrease risk of serious clinical events with maximal generalizability and statistical power.

To test this approach, we undertook a systematic examination of all potentially existing associations between prescribed drugs and short-term risk for AMI using comprehensive nation-wide data from Sweden and Norway. Given the very strong likelihood of confounding by indication in pharmacoepidemiology, we focused on short-term AMI risk and used case-only methods for self-matching. Because this approach may overestimate the effects of drugs used chronically^7^, it yields estimates that are most reliable for drugs typically taken for short time periods (for acute use).

## Results

Among a total of 121,652 AMI patients, 97,068 were from Sweden (79,882 and 17,186 identified via the patient register and the cause of death register, respectively) and 24,584 were from Norway (20,413 and 4,171 identified via the patient register and the cause of death register, respectively). Characteristics of these patients are presented in Table 1.

**Table 1:** Characteristics of the study sample.

Characteristics	Total N (%)	Sweden N (%)	Norway N (%)
Total AMI patients*	121, 652	97,068	24,584
Males	65,171 (53.6)	50,583 (52.1)	14,588 (59.3)
**Age (in years)**			
Below 30	65 (0.1)	43 (0.04)	22 (0.1)
30–39	428 (0.4)	256 (0.3)	172 (0.7)
40–49	2836 (2.3)	1785 (1.8)	1051 (4.3)
50–59	9299 (7.6)	6369 (6.6)	2930 (11.9)
60–69	20,597 (16.9)	15,434 (15.9)	5163 (21)
70–79	30,710 (25.2)	24,539 (25.3)	6171 (25.1)
80–89	43,126 (35.5)	36,113 (37.2)	7013 (28.5)
90–99	14,419 (11,9)	12,381 (12.8)	2038 (8.3)
≥100	172 (0.1)	148 (0.2)	24 (0.1)

*The number reflects the patients who were hospitalized or died due to acute myocardial infarction both within and outside hospital.

In addition, the numbers reflects the patients who dispensed prescribed drugs either in the case-period (1 to 7 days) or control-period (15 to 21 days) before the index date for the diagnosis of their AMI in Norway and Sweden, respectively.

**Figure 1:**
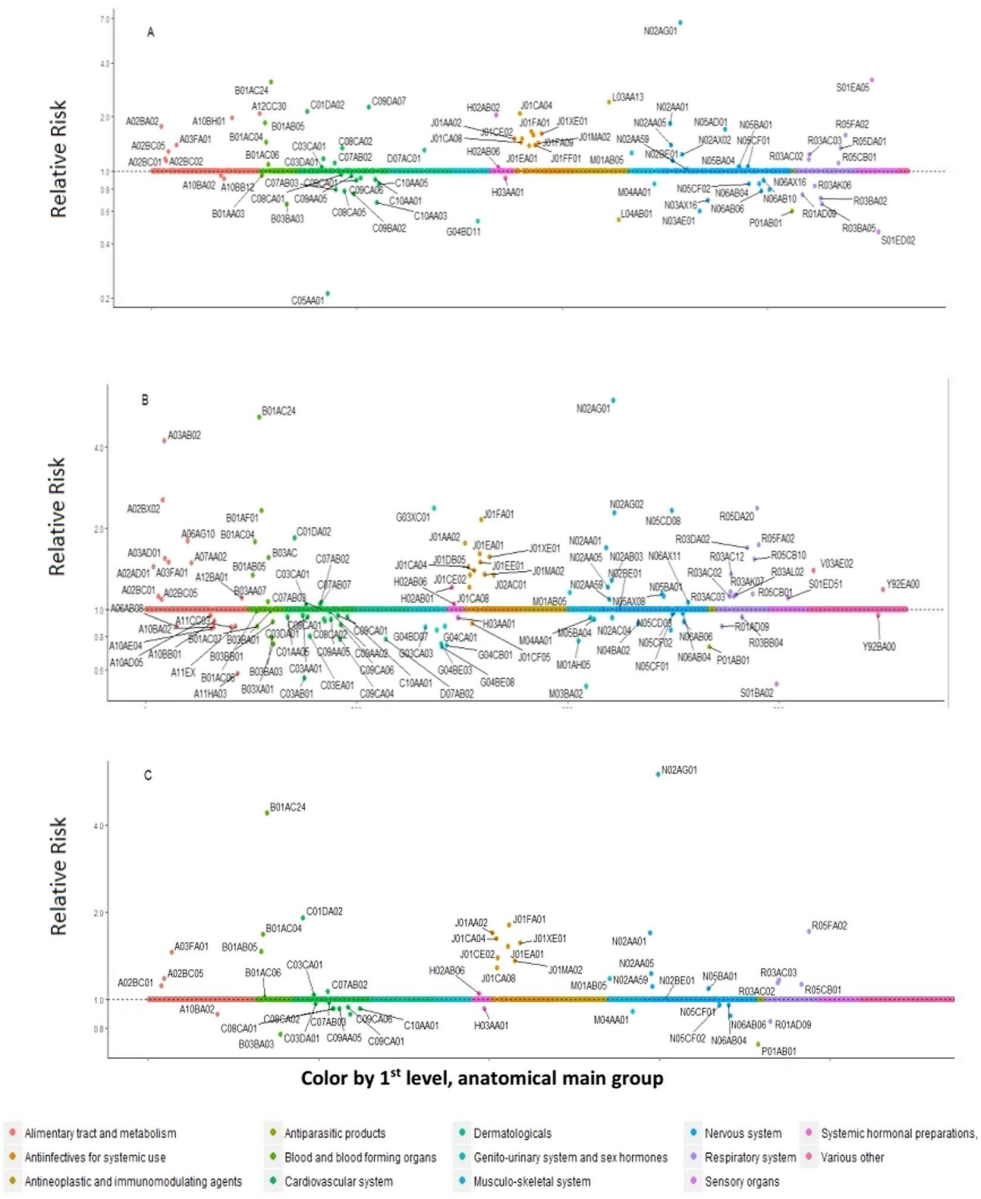
Pharmacopeia-wide case-crossover analysis of dispensed medication data in relation to risk for acute myocardial infarction. The above plot illustrates (**A**) 81 unique drug types which were selected in Norway, (**B**) 112 unique drug types which were selected in Sweden, and (**C**) 48 drugs which were common hits from both the countries. Y-axis displays relative risk on the log scale, X-axis displays all the prescribed drugs studied grouped according to the Anatomical Therapeutic Chemical (ATC) classification.

A total of 1262 and 1535 distinct pharmaceutical drugs were dispensed for AMI patients in Norway and Sweden, respectively. Out of these, 737 and 1081 unique drugs were dispensed for AMI patients in either the case- or control- period, in Norway and Sweden, respectively. Subsequently, a total of 81 and 112 unique drug types were selected in Norway and Sweden via BOLASSO procedure, respectively. Finally, a total of 48 drugs were selected in common from both countries (Figure 1). Table 2 presents the country-specific and the combined estimates of these commonly selected drugs.

**Table 2:** Relative risks of acute myocardial infarction within 7 days after the drug was dispensed, selected by BOLASSO approach^†^ in both countries.

ATC Code^Ѕ^	Drug Names	Exposed Case- period only	Exposed Control- period only	Sweden^‡^	Exposed Case- period only	Exposed Control- period only	Norway^§^	Total^‖^
				RR (95% CI)			RR (95% CI)	Combined estimates RR (95% CI)
***Narcotic analgesics***								
N02AG01	Morphine & antispasmodics	256	42	5.92 (4.09–8.57)	28	2	6.66 (2.31–19.36)	6.00 (4.23–8.50)
N02AA01	Morphine	2195	1515	1.69 (1.52–1.89)	137	67	1.84 (1.21–2.79)	1.70 (1.53–1.89)
N02AA05	Oxycodone	2659	2337	1.21 (1.10–1.34)	252	191	1.40 (1.07–1.84)	1.23 (1.12–1.35)
N02AA59	Codeine, combinations	1526	1391	1.09 (1.00–1.20)	1386	1202	1.14 (1.03–1.25)	1.11 (1.04–1.19)
R05FA02	Opium derivatives & expectorant	828	438	1.73 (1.52–1.97)	71	35	1.59 (1.00–2.54)	1.72 (1.52–1.95)
***Non-narcotic analgesic***								
N02BE01	Paracetemol	14481	14036	1.01 (0.75–1.05)	1412	1290	1.02 (0.93–1.12)	1.01 (0.98–1.05)
***Anti-inflammatory drug***								
M01AB05	Diclofenac	1180	1001	1.15 (1.04–1.28)	504	387	1.26 (1.07–1.47)	1.18 (1.08–1.29)
***Antibiotics***								
J01FA01	Erythromycin	90	36	2.14 (1.39–3.30)	156	89	1.66 (1.22–2.24)	1.81 (1.41–2.32)
J01AA02	Doxycycline	1095	591	1.76 (1.58–1.96)	324	213	1.51 (1.25–1.83)	1.70 (1.54–1.86)
J01CA04	Amoxicillin	575	353	1.44 (1.24–1.66)	327	168	2.09 (1.69–2.58)	1.62 (1.44–1.83)
J01XE01	Nitrofurantoin	365	249	1.56 (1.29–1.89)	105	66	1.62 (1.12–2.33)	1.57 (1.33–1.86)
J01EA01	Trimethoprim	402	281	1.60 (1.33–1.92)	149	114	1.38 (1.03–1.84)	1.53 (1.31–1.79)
J01CE02	Phenoxymethylpenicillin	847	595	1.34 (1.20–1.50)	423	277	1.51 (1.29–1.77)	1.39 (1.27–1.53)
J01MA02	Ciproflolaxin	830	630	1.35 (1.19–1.52)	194	134	1.43 (1.10–1.86)	1.36 (1.22–1.52)
J01CA08	Pivmecillinam	535	422	1.21 (1.05–1.41)	309	211	1.45 (1.19–1.78)	1.29 (1.14–1.45)
***Antiprotozoal drug***								
P01AB01	Metronidazole	169	185	0.73 (0.57–0.93)	51	51	0.60 (0.38–0.94)	0.70 (0.56–0.87)
***Antithrombotic agents***								
B01AC24	Ticagrelor	96	14	5.14 (2.85–9.26)	27	6	3.12 (1.27–7.67)	4.42 (2.70–7.24)
B01AC04	Clopidogrel	1290	852	1.78 (1.57–2.01)	391	251	1.44 (1.19–1.76)	1.68 (1.51–1.86)
B01AB05	Enoxaparin	126	93	1.34 (0.98–1.83)	78	43	1.85 (1.14–3.00)	1.47 (1.13–1.92)
B01AC06	Acetylsalicylic acid	15489	15240	0.99 (0.95–1.03)	3141	2803	1.09 (1.02–1.17)	1.02 (0.98–1.05)
***Antiadrenergic agents***								
R03AC03	Terbutaline	968	787	1.16 (1.04–1.30)	92	77	1.24 (0.89–1.73)	1.17 (1.05–1.30)
R03AC02	Salbutamol	618	526	1.12 (0.98–1.28)	653	532	1.16 (1.00–1.34)	1.14 (1.03–1.26)
***Proton pump inhibitors***								
A02BC05	Esomeprazol	871	802	1.09 (0.96–1.25)	725	588	1.29 (1.13–1.48)	1.18 (1.08–1.30)
A02BC01	Omeprazole	8870	8261	1.12 (1.07–1.18)	238	213	1.17 (0.94–1.45)	1.12 (1.07–1.18)
***Vasodilator***								
C01DA02	Glyceral trinitrate	5020	2949	1.84 (1.74–1.94)	1758	864	2.15 (1.96–2.36)	1.92 (1.83–2.01)
***Prokinetic drug***								
A03FA01	Metoclopramide	709	503	1.49 (1.28–1.73)	335	220	1.40 (1.12–1.75)	1.46 (1.29–1.66)
***Expectorant***								
R05CB01	Acetylcysteine	1677	1375	1.14 (1.05–1.24)	519	394	1.11 (0.96–1.30)	1.13 (1.05–1.22)
***Anxiolytic***								
N05BA01	Diazepam	1165	957	1.12 (0.99–1.26)	664	575	1.06 (0.93–1.22)	1.09 (1.00–1.20)
***Angiotension II antagonists***								
C09CA01	Losartan	1728	1738	0.95 (0.86–1.05)	292	310	0.89 (0.73–1.08)	0.94 (0.86–1.02)
C09CA06	Candesartan	1363	1448	0.88 (0.79–0.98)	360	387	0.92 (0.77–1.09)	0.89 (0.81–0.98)
***Antidepressants***								
N06AB04	Citalopram	5548	5513	0.97 (0.90–1.05)	170	182	0.85 (0.66–1.11)	0.96 (0.89–1.03)
N06AB06	Sertraline	1358	1360	0.90 (0.78–1.04)	90	98	0.78 (0.53–1.13)	0.88 (0.77–1.01)
***ACE inhibitor***								
C09AA05	Ramipril	1870	1855	0.92 (0.83–1.02)	709	683	0.94 (0.81–1.08)	0.93 (0.85–1.01)
***Antigout agent***								
M04AA01	Allopurinol	1897	1952	0.93 (0.83–1.03)	351	394	0.85 (0.70–1.04)	0.91 (0.83–1.00)
***Antidiabetic agent***								
A10BA02	Metformin	2659	2896	0.86 (0.79–0.94)	693	695	0.95 (0.83–1.09)	0.89 (0.82–0.95)
***Beta-blocking agents***								
C07AB02	Metoprolol	8861	8437	1.05 (1.00–1.11)	2579	2289	1.11 (1.02–1.20)	1.07 (1.02–1.12)
C07AB03	Atenolol	2836	2823	1.01 (0.93–1.09)	252	285	0.79 (0.65–0.96)	0.98 (0.91–1.05)
***Calcium-channel blockers***								
C08CA01	Amlodipine	2512	2544	0.92 (0.85–1.00)	706	718	0.96 (0.85–1.09)	0.93 (0.87–1.00)
C08CA02	Felodipine	3529	3617	0.91 (0.85–0.98)	112	93	1.34 (0.96–1.87)	0.93 (0.86–0.99)
***Decongestant***								
R01AD09	Mometasone	326	338	0.87 (0.73–1.03)	83	100	0.74 (0.54–1.03)	0.84 (0.72–0.98)
***Diuretics***								
C03CA01	Furosemide	14307	13807	1.04 (0.99–1.09)	1317	1201	1.05 (0.95–1.17)	1.04 (1.00–1.09)
C03DA01	Spironolactone	2716	2674	0.94 (0.86–1.03)	258	219	1.17 (0.92–1.48)	0.97 (0.89–1.05)
***Glucocorticoid***								
H02AB06	Prednisolone	3692	3486	1.05 (0.97–1.13)	963	823	1.05 (0.94–1.17)	1.05 (0.99–1.12)
***Hypnotics and sedatives***								
N05CF01	Zopiclone	5999	6024	0.96 (0.90–1.02)	1618	1460	1.06 (0.97–1.17)	0.99 (0.94–1.04)
N05CF02	Zolpidem	2555	2564	0.97 (0.89–1.05)	175	191	0.85 (0.67–1.07)	0.96 (0.88–1.03)
***Lipid modifying agent***								
C10AA01	Simvastatin	5611	5672	0.94 (0.88–0.99)	1806	1748	0.90 (0.82–0.98)	0.93 (0.88–0.97)
***Antithyroid agent***								
H03AA01	Levothyroxine sodium	4843	4863	0.93 (0.86–1.01)	629	648	0.92 (0.80–1.06)	0.93 (0.87–1.00)
***Antianemic agent***								
B03BA03	Hydroxocobalamin	125	155	0.79 (0.61–1.01)	49	63	0.66 (0.43–1.02)	0.76 (0.61–0.94)

*Case crossover analysis, case period (1 to 7 days) and control period (15 to 21 days) before the index-date for the diagnosis of AMI.

^†^In BOLASSO, the effect of each selected drugs is controlled for the effect of other selected drugs.

^‡^Data from the Swedish Prescribed Drug Register, 2005 to 2014.

^§^Data from the Norwegian Prescription Database, 2004 to 2014.

^‖^Combined estimates of Norwegian and Swedish data calculated using fixed-effect model.

^S^Classified according to Anatomical Therapeutic Chemical (ATC), 5^th^ level.

### Cardiovascular drugs

Several cardiovascular drugs were associated with higher AMI risk, potentially related to their indications for use. These medications included nitroglycerin and different classes of antithrombotic agents (ticagrelor, clopidogrel, enoxaparin, acetylsalicylic acid). On the other hand, certain angiotensin-II-antagonists (losartan, candesartan), calcium-channel blockers (amlodipine, felodipine), and an angiotensin-converting-enzyme inhibitor (ramipril) were associated with lower risk of AMI.

### Antibiotics

Use of penicillins, ciprofloxacin, doxycycline, erythromycin, trimethoprim and nitrofurantoin were associated with higher risk of AMI. In contrast, metronidazole was associated with lower risk of AMI.

### Analgesics

Several opioid analgesics were associated with higher risk of AMI. Of these, morphine in combination with antispasmodics was associated with particularly high risk for AMI. Among non-opioid agents, diclofenac was also associated with increased risk for AMI.

### Psychoactive medications

Among anxiolytic, antidepressant, and antipsychotic agents, we observed an increased AMI risk in association with the use of diazepam. Two serotonin-specific reuptake inhibitors, citalopram and sertraline, were associated with lower risk for AMI.

### Other medications

Specific adrenergic agents (terbutaline, salbutamol), proton pump inhibitors (esomeprazole and omeprazole), metoclopramide and acetylcysteine were associated with higher risk of AMI. On the other hand, allopurinol, mometasone, metformin, simvastatin and levothyroxine were inversely associated with risk of AMI.

In online supplementary material, Tables S1 and S2, we present all drugs selected by BOLASSO in either Norway or Sweden, respectively. Apart from the results of the BOLASSO, we also present the univariable, non-penalized estimates for the selected drugs in these tables.

### Sensitivity analysis

In online Supplementary Table S3, we present the results of our analyses where we extended the case-, control- and wash-out periods from 7 days to 14 days. Point estimates were generally similar to those in our main analyses. However, these analyses selected slightly more drugs. Some additional cardiovascular drugs (vasodilator isosorbide mononitrate, diuretic bendroflumethiazide, beta-blocker bisoprolol) were associated with higher AMI risk; whereas others (angiotensin II antagonist valsartan, calcium channel blocker amlodipine, pravastatin, atorvastatin and diuretic spironolactone) were associated with lower risk. Some additional antibiotics (clindamycin and nystatin), opioid analgesics (ketobemidone in combination with antispasmodics, fentanyl, tramadol), the anti-cancer drug capecitabine, the anxiolytic oxazepam and the antidepressant mirtazapine were also associated with increased risk. For norethisterone and estrogen, tolterodine, hydrocortisone, budesonide, tiotropium bromide, salmeterol and fluticasone, we observed lower risk in the extended analyses.

In online Supplementary Table S4, we present analyses restricted to those AMI cases diagnosed in hospital. The results were largely similar to the main analyses, but BOLASSO selected fewer drugs, possibly due to the lower statistical power.

In online Supplementary Tables S5 and S6, we present sub-group analyses by age (dichotomized at 80 years) for 48 drugs selected via BOLASSO approach in both countries. Relative risks were generally comparable between the two age groups.

## Discussion

In this pharmacopeia-wide association study, we investigated all possible associations between pharmaceutical drugs requiring a prescription and short-term risk for AMI using nationwide data from two countries, Norway and Sweden. We identified 48 drugs that were either associated with increase or decrease in short-term risk for AMI in both countries.

Our approach had similarities with genome-wide association studies, but also some important differences that are reflected in our analytic strategy. Bonferroni or similar adjustments are widely used in genome-wide association studies to handle the problem of multiple comparisons. These methods adjust the *p* value or the significance level based on the number of tests performed and decrease the occurrence of false positive findings, albeit at the expense of increasing the occurrence of false negative findings. The Bonferroni method also assumes that associations are essentially random and tests a joint null hypothesis that no exposures are associated with the outcome. This might be justifiable for genome-wide association studies, where, at most, only a handful of true associations are likely out of millions of tested candidates. However, such a joint null hypothesis makes less sense in our study, because many medications clearly do influence AMI risk^8^. Finally, and most importantly, Bonferroni and related methods can increase the rate of true positive findings but cannot improve estimation. On the contrary, estimates for effects selected based on Bonferroni adjusted significance are likely to be substantially inflated. In contrast, penalized regression can greatly improve the accuracy of estimation over conventional methods in the setting of multiple comparisons^9,10^.

It may be useful to consider the medications that we found to be associated with AMI risk in several categories, based upon standard epidemiological principles. In contrast to GWAS, PWAS examines medications that vary frequently in time and bear specific indications, and hence are prone to confounding and other issues for which appropriate epidemiological cautions are necessary. First are cases in which reverse causation may be at play. This includes drugs specifically used to treat accelerating symptoms of myocardial ischemia such as analgesics, nitroglycerin or metoprolol, but also drugs used to treat conditions that produce chest pain or other ischemic symptoms, such as gastroesophageal reflux, anxiety attacks or respiratory conditions. This latter could explain the observed associations for proton pump inhibitors, antiadrenergic agents, and anxiolytics.

One approach to address reverse causation is comparison within a single drug class, where members usually have similar if not identical indications. Large differences between the observed associations for drugs within the same class might indicate true differences in cardiovascular effects. For example, morphine in combination with antispasmodics had a much stronger association with short-term risk of AMI than other opioids, suggesting that this combination could be a strong candidate for further in-depth studies that may eventually result in regulatory changes. Also, ticagrelor had a considerably stronger association with AMI risk than other antithrombotic agents, although this may reflect its narrower range of use, namely that is typically prescribed in the acute phases before and after AMI when the risk of recurrence is highest.

Second are cases of probable confounding. This particularly affects drugs used to treat conditions that themselves increase risk of AMI, such as antibiotics to treat infections and drugs to treat inflammatory or acute painful conditions that may increase risk similarly. Although these associations are unlikely to be causal, PWAS offers the particular advantage that it may, by proxy, identify important physiological pathways leading to AMI. Although infection has been associated with triggering of AMI in previous studies^11^, our much larger study provides a richer source of evidence for this hypothesis. Much like reverse causation, comparison within class, even for confounders, may yield important insights, and our results suggest that metronidazole may acutely lower risk of AMI. Suggestively, this may reflect the disulfiram-like activity of metronidazole, given the association of acute alcohol intake with AMI^12^ and the absence of other commonly-used drugs that inhibit aldehyde dehydrogenase activity. Alternatively, animal and *in vitro* studies have suggested that pathogens such as helminths might protect against atherosclerosis via activation of the Th2 pathway^13,14^. This could imply that the association of metronidazole with a lower risk of AMI might reflect a cardioprotective effect of an underlying parasitic infection. However, given the broad range of indications for metronidazole, further study of this possibility is needed.

Third are true associations in which drugs may causally influence AMI risk. This group includes drugs that plausibly increases or decreases risk themselves and, equally, in which withdrawal of an existing medication increases risk. The latter mechanism is the most plausible explanation for the inverse association with AMI for statins, calcium-channel blockers, angiotensin II antagonists and angiotensin-converting-enzyme inhibitors. Our results suggest that clinicians should exercise caution in withdrawing these medications to avoid rebound increases in risk. The inverse associations between AMI risk and some antidepressants, allopurinol and metformin were also expected based on prior epidemiological studies^15–18^ and the known pathophysiological effects of these drugs. For example, SSRIs possess acute anti-platelet effects^19^, allopurinol seems to reduce oxidative stress, to prevent plaque instability and to have anti-ischemic properties^20^ and metformin may improve endothelial function^21^. However, our results provide large-scale evidence of associations that extend to triggering of clinical events.

Lastly, PWAS has the potential to identify new, unexpected agents, where the mechanism of action is uncertain but that clearly require further study. The inverse associations between AMI risk and use of hydroxocobalamin, levothyroxine and mometasone are examples for such unexpected and unexplained findings in the present study. We are not aware of earlier studies suggesting acute cardioprotection for these drugs; neither can these associations be readily explained by the indication nor by the known effects of these drugs. It is possible that they are markers of clinical stability and only prescribed when patients are at particularly low risk, but further clinical detail would be needed to explore this possibility.

To the best of our knowledge, only two studies, one from the United States^22^ and another from Europe^23^ have adopted somewhat similar approaches and investigated the association between drugs and AMI in a hypothesis-free manner. In contrast to our study, the data from these prior studies were not nationwide, but relied on databases of administrative claims^24^, and the endpoint of AMI was not based on a clinical diagnosis in a hospital. None of the studies applied a case-crossover design. Furthermore, these studies used Bonferroni adjustment for multiple comparisons. Thus, the effect sizes were not comparable with those in our study. However, overall, the group of drugs selected as being associated with AMI risk in these two earlier studies were generally similar to those in our study, further supporting the value of this approach as a reproducible method potentially appropriate for standardization, automation, and continuous monitoring of sentinel events within national health systems. In this regard, our approach fits within the rubric of adaptive biomedical innovation, which seeks to apply real-time, large-scale, rapid-cycle clinical data to the process of approving, monitoring, and targeting new pharmaceuticals, devices, and other forms of therapy^25^.

### Strength and limitations

We conducted a nation-wide study in two countries where health care systems are universal and equally accessible to virtually all the residents. Reporting is mandatory in the patient registers, cause of death registries and prescription databases in both Norway and Sweden. Therefore, our study is not subject to bias due to self-selection or to recall bias. Validation studies confirm that the quality of information in the Norwegian and Swedish Patient Registers is very high on AMI^26,27^. Furthermore, the prescription databases in both countries are fully digitalized, and hence little chance of misclassification of drug retrieval exists. Due to the case-crossover design, our results are unlikely to be confounded by stable patient characteristics, chronic conditions or lifestyle related factors predisposing to AMI and influencing medication use^28^, although they are prone to confounding by characteristics that fluctuate within individuals over days-to-weeks.

Apart from these strengths, our study also has important limitations. Although we used a BOLASSO to minimize the problem of multiple comparisons and relied on nation-wide data from two countries, but nonetheless we cannot rule out the possibility that some associations are due to chance and further studies are needed to confirm the findings from this large-scale screening. This limitation is common to all broad-based, agnostic screening approaches. Similar to GWAS, our study should be regarded as a first step in monitoring the pharmacopoeia, with direct clinical impact occurring later. For example, the first GWAS on age-related macular degeneration was conducted in 2005^29^, and it eventually led to promising therapies for age-related macular degeneration in 2016^30^.

Given the exploratory nature of our work, we analyzed all drugs in a uniform way and consequently the hypothesized case-, control- and wash-out periods might not be optimal for several drugs. It is important to recognize that such uncertainties lead to a bias towards null in a case-crossover study^28,31^. Thus, under- but not overestimation of effects is likely in case of some drugs due to the suboptimal length of time windows. It is important to emphasize, however, that when we extended the case-, control- and wash-out periods in our sensitivity analyses, we observed generally similar results. Also, case-crossover studies are prone to the so-called ‘persistent user bias’^7^ which might lead to an upward bias of the estimates in case of chronically-used drugs. As this study lacks data to distinct between medications for acute use and chronic use. Thus, these results must be interpret cautiously on clinical grounds; particularly those prescribed drugs that are used chronically. However, it cannot explain the novel findings in this study, such as the observed protective effect of hydroxocobalamin, levothyroxine and mometasone, nor to differences within class. Also, given the register-based nature of our study, we do not have additional information on patient characteristics such as cardiovascular risk factors or previous cardiac events, although our use of the case-crossover design precludes confounding by stable personal characteristics.

Finally, Norwegian Prescription Database and Swedish Prescribed Drug Register contain complete and valid information on dispensed drugs, but they do not describe the actual date of self-administration. This most probably led to a non-differential misclassification and a bias toward null findings. In this regard, claims-based datasets may have potential advantages for extending and refining this approach.

In summary, this pharmacopeia-wide association study demonstrates the feasibility and results of a systematic, unbiased approach to pharmacological triggers of AMI. Our results also highlight the value of rigorous epidemiological consideration of observed findings. This approach also offers the possibility of routine, rigorous monitoring of the worldwide pharmacopeia for agents that trigger, or prevent the triggering of, serious acute health events.

## Methods

### Study design

We applied a case-crossover design^28,31^, which includes only cases of a disease and applies self-matching by comparing exposure before the disease onset with disease-free time in the past as control information. This design is a special case of the conventional matched case-control design, and it has common features with cross-over trials. A primary advantage of the case-crossover design is that stable within-person characteristics cannot confound observed associations, although time-varying features can. Today, it is largely considered a standard method to study acute or triggering effects of transient exposures on serious outcomes such as AMI^32–34^.

### Ascertainment of AMI

We used the Norwegian Patient Registry, the Swedish National Patient Registry, and cause of death registries in Norway and Sweden to identify AMI cases^35,36^. Validation studies show that the quality of information on AMI in these registers, especially in the Norwegian Patient Registry and the Swedish National Patient Register is very high^26,27^. In Norway, all patients with primary ICD-10 hospital discharge diagnoses of I21 or I22 from 1 January 2008 to 31 December 2014 were included, as were individuals with a comparable cause of death from 1 January 2004 to 31 December 2014. In Sweden, the corresponding dates for both the hospital diagnosis and cause of death were between 1^st^ November 2005 and 31^st^ December 2014. For each individual, only the first registered episode of AMI was included in the analyses.

### Prescribed medications

We assessed the risk of AMI associated with each and every drug prescribed to AMI patients. Data on dispensed medications prior to the occurrence of AMI were abstracted from the nation-wide registration of dispensed drugs in Norway and Sweden, respectively. The Norwegian Prescription Database was established in 2004^37^. All Norwegian pharmacies are required to supply information from prescriptions including type and dosage of the drug and date of drug dispense. Sweden has also established a similar register, the Swedish Prescribed Drug Register in 2005^38^. A personal identifier is attached to these data that makes it possible to link the information on drug use to other health related registers existing in these countries. The prescription databases do not include information on drugs purchased over-the-counter, or given to institutionalized patients in nursing homes or hospitals. In Norway, it was possible to exclude participants who, at the time of AMI, were institutionalized and for whom registration of dispensed medications was not available. In Sweden, in the absence of this information, we included only those patients who had at least one drug prescribed during one year before the occurrence of AMI.

### Statistical analyses

For each patient, the occurrence of drug dispensing within 1 to 7 days before the date of diagnosis of AMI (case period) was compared to a time window of 15 to 21 days before the AMI diagnosis (control period) for each drug individually. To minimize the carryover effects of drugs, we included a one-week wash-out period between the case- and the control-periods. We calculated odds ratios together with 95% confidence intervals, comparing the odds of drug dispensed in the case period to that in the control period using conditional logistic regression.

We assessed all prescribed medications in relation to AMI risk. Because our aim was to identify drugs with a real effect and to assess the size of these effects, we opted not to use Bonferroni adjustment and similar conventional methods to address the problem of multiple comparisons inherent to such settings, as they fail to estimate the size of these associations correctly^10^. Instead, we applied a version of the least absolute shrinkage and selection operator (LASSO) regression analysis^9,10,39–41^ called BOLASSO (bootstrap-enhanced least absolute shrinkage operator)^42^. With the BOLASSO, a number of bootstrap samples are drawn from the dataset, where each bootstrap sample is generated by sampling N pairs (N is the total number of drugs in the dataset) with replacement. Here, we have drawn 1000 bootstrap samples. Of note, confidence intervals generated via BOLASSO approach are not optimal, because each bootstrap sample is estimated on different penalty parameters. Therefore, some of the drugs selected by this approach may include one in their confidence interval. In online supplementary material, Appendix A, we present in detail the background of the method and how we implemented BOLASSO in conditional logistic regression models for case-crossover data.

We conducted separate analyses for Norwegian and Swedish data. We presented both country-specific and combined estimates for drugs selected by BOLASSO from both countries. In BOLASSO, the effect of each selected drug is controlled for the effects of all other selected drugs. The combined estimates were calculated using fixed-effect models^43^.

We performed sensitivity analyses to examine the robustness of our results. Firstly, we extended the case-, control- and wash-out periods from 7 days to 14 days (case period = 1 to 14 days; control period = 29 to 42 days) and repeated all analyses. Secondly, because a diagnosis of AMI is likely to be more reliable when given at a hospital than when provided as a cause of death, we also repeated our main analyses when we excluded patients who died outside of hospital. Lastly, we performed subgroup analyses by age (dichotomized at 80 years) on drugs selected by BOLASSO approach in both the countries. Separate analyses were performed for Norwegian and Swedish data.

All statistical analyses were performed using R (version 3.2.3; R foundation for Statistical Computing, Vienna, Austria) and Stata/IC 14 (Stata Corp, College Station, Texas, USA).

### Ethical approval and informed consent

Informed consent for study participation was obtained by the national registers from the respective country. The data for the patients from the respective registers was send to us anonymously. Thus, in our present research, no patients were involved in setting the research question or the outcome measures, nor were they involved in developing plans for recruitment, design, or implementation of the study. No patients were asked to advise on interpretation or writing up of results.

The studies were approved by the Regional Committees for Medical and Health Research Ethics (REC) in Central Norway and Regional Ethical Review Board in Sweden. In addition, Norwegian data was also approved by Norwegian Data Protection Authority (Datatilsynet). All methods were performed in accordance with the relevant guidelines and regulations by the respective ethical committees from both Norway and Sweden.

## Supplementary information

Supplementary Tables and Appendix

## Data Availability

The data that support the findings of this study are available from the Norwegian Patient Registry, Norwegian Prescription database, Norwegian Cause of Death Registry, and Swedish National Patient Registry, Swedish Prescribed Drug Registry, Swedish Cause of Death Registry but restrictions apply to the availability of these data, which were used under license for the current study, and so are not publicly available. Data are however available from the corresponding authors upon reasonable request and with permission of respective national registry in Norway and Sweden.
