# Author Correction: Systematic assessment of prescribed medications and short-term risk of myocardial infarction – a pharmacopeia-wide association study from Norway and Sweden

**DOI:** 10.1038/s41598-020-57847-5

**Published:** 2020-02-04

**Authors:** Abhijit Sen, Ioannis Vardaxis, Bo Henry Lindqvist, Ben Michael Brumpton, Linn Beate Strand, Inger Johanne Bakken, Lars Johan Vatten, Pål Richard Romundstad, Rickard Ljung, Kenneth Jay Mukamal, Imre Janszky

**Affiliations:** 1 Department of Public health and Nursing, Faculty of Medicine and Health Sciences, Norwegian University of Science and Technology, 7491 Trondheim, Norway; 2 Center for Oral Health Services and Research (TkMidt), Trondheim, Norway; 3 Department of Mathematical Sciences, Norwegian University of Science and Technology, 7491 Trondheim, Norway; 4 Department of Thoracic Medicine, St. Olav’s Hospital, Trondheim University Hospital, Trondheim, Norway; 5 K.G. Jebsen Centre for Genetic Epidemiology, Department of Public Health and Nursing, Norwegian University of Science and Technology, 7491 Trondheim, Norway; 6 MRC Integrative Epidemiology Unit, University of Bristol, Bristol, United Kingdom; 7 Centre for Fertility and Health (CeFH), Norwegian Institute of Public Health, Oslo, Norway; 8 Unit of Epidemiology, Institute of Environmental Medicine, Karolinska Institutet, SE 171 77, Solna, Stockholm, Sweden; 9 Department of Medicine, Beth Israel Deaconess Medical Center, Boston, MA United States; 10 Regional Center for Health Care Improvement, St Olav’s Hospital, Trondheim, Norway

Correction to: *Scientific Reports* 10.1038/s41598-019-44641-1, published online 4 June 2019

In the Supplementary Information file originally published with this Article, Appendix A was omitted. This error has been corrected in the Supplementary Information that now accompanies the Article.

