# Supplementary material for: Systematic assessment of prescribed medications and short-term risk of myocardial infarction – a pharmacopeia-wide association study from Norway and Sweden
Source: Sci Rep. 2019 Jun 4;9:8257. doi: 10.1038/s41598-019-44641-1 (PMC6547702; doi:10.1038/s41598-019-44641-1)
Supplement: Supplementary file 1 — Supplementary Tables and Appendix [file 41598_2019_44641_MOESM1_ESM.pdf]

Supplementary file

Title: Systematic assessment of prescribed medications and short-term risk of myocardial infarction – a pharmacopeia-wide association study from Norway and Sweden

Abhijit Sen*, PhD[1,2], Ioannis Vardaxis, MSc[3], Bo Henry Lindqvist, PhD[3], Ben Michael Brumpton, PhD[4,5,6], Linn Beate Strand, PhD[1], Inger Johanne Bakken, PhD[7], Lars Johan Vatten, MD, PhD[1], Pål Richard Romundstad, PhD[1], Rickard Ljung, MD PhD[8], Kenneth Jay Mukamal, MD[9], Imre Janszky, MD PhD[1,10]

Affiliations

[1]Department of Public health and Nursing, Faculty of Medicine and Health Sciences, Norwegian University of Science and Technology, 7491 Trondheim, Norway.
[2]Center for Oral Health Services and Research (TkMidt), Trondheim, Norway
[3]Department of Mathematical Sciences, Norwegian University of Science and Technology, 7491 Trondheim, Norway.
[4]Department of Thoracic Medicine, St. Olav's Hospital, Trondheim University Hospital, Trondheim, Norway.
[5]K.G. Jebsen Centre for Genetic Epidemiology, Department of Public Health and Nursing, Norwegian University of Science and Technology, 7491 Trondheim, Norway.
[6]MRC Integrative Epidemiology Unit, University of Bristol, UK.
[7]Centre for Fertility and Health (CeFH), Norwegian Institute of Public Health, Oslo, Norway.
[8]Unit of Epidemiology, Institute of Environmental Medicine, Karolinska Institutet, SE 171 77, Solna, Stockholm, Sweden.
[9]Department of Medicine, Beth Israel Deaconess Medical Center, Boston, MA, United States.
[10]Regional Center for Health Care Improvement, St Olav's Hospital, Trondheim, Norway

***Correspondence:**

Abhijit Sen, PhD
Email: abhijit.sen@ntnu.no

Table S1 Relative risks of acute myocardial infarction within 7 days after the drug was dispensed. Univariable and BOLASSO results for drugs selected by BOLASSO approach in Norway.

Table S2 Relative risks of acute myocardial infarction within 7 days after the drug was dispensed. Univariable and BOLASSO results for drugs selected by BOLASSO approach in Sweden.

Table S3 Relative risks of acute myocardial infarction within 14 days after the drug was dispensed, selected by BOLASSO approach in both countries.

Table S4 Relative risks of acute myocardial infarction within 7 days after the drug was dispensed, selected by BOLASSO approach in both countries. AMI diagnosed in hospital only.

Table S5 Relative risks of acute myocardial infarction within 7 days after the drug was dispensed. Univariable results for drugs selected by BOLASSO approach in both the countries. Sub-group analyses according to age in Norway

Table S6 Relative risks of acute myocardial infarction within 7 days after the drug was dispensed. Univariable results for drugs selected by BOLASSO approach in both the countries. Sub-group analyses according to age in Sweden

Table S1. Relative risks of acute myocardial infarction within 7 days after the drug was dispensed [a]. Univariable and BOLASSO results for drugs selected by BOLASSO approach in Norway[b]

ATC[d]	Drug Name	Case window (N exposed) /Control window (N exposed)	Univariable	BOLASSO[c]
			RR (95% CI)	RR (95% CI)
**A02BA02**	Ranitidine	239 / 143	1.67 (1.36 to 2.06)	1.76 (1.37 to 2.26)
**A02BC01**	Omeprazole	238 / 213	1.12 (0.93 to 1.34)	1.17 (0.94 to 1.45)
**A02BC02**	Pantoprazole	662 / 590	1.12 (1.00 to 1.25)	1.14 (1.00 to 1.31)
**A02BC05**	Esomeprazole	725 / 588	1.23 (1.11 to 1.37)	1.29 (1.13 to 1.48)
**A03FA01**	Metoclopramide	335 / 220	1.52 (1.38 to 1.81)	1.40 (1.12 to 1.75)
**A10BA02**	Metformin	693 / 695	0.99 (0.90 to 1.11)	0.95 (0.83 to 1.09)
**A10BB12**	Glimepiride	341 / 349	0.98 (0.84 to1.13)	0.91 (0.76 to 1.09)
**A10BH01**	Sitagliptin	35 / 18	1.94 (1.10 to 3.43)	1.98 (0.99 to 3.98)
**A12CC30**	Magnesium	39 / 24	1.63 (0.98 to 2.70)	2.09 (0.94 to 4.62)
**B01AA03**	Warfarin	489 / 493	0.99 (0.88 to 1.12)	0.95 (0.83 to 1.09)
**B01AB05**	Enoxaparin	78 / 43	1.81 (1.25 to 2.63)	1.85 (1.14 to 3.00)
**B01AC04**	Clopidogrel	391 / 251	1.56 (1.33 to 1.83)	1.44 (1.19 to 1.76)
**B01AC06**	Acetylsalicylic acid	3141 / 2803	1.12 (1.06 to 1.18)	1.09 (1.02 to 1.17)
**B01AC24**	Ticagrelor	27 / 6	4.50 (1.86 to 10.9)	3.12 (1.27 to 7.67)
**B03BA03**	Hydroxocobalamin	49 / 63	0.78 (0.54 to 1.13)	0.66 (0.43 to 1.02)
**C01DA02**	Glyceral trinitrate	1758 / 864	2.03 (1.88 to 2.21)	2.15 (1.96 to 2.36)
**C01DA14**	Isosorbide mononitrate	1072 / 955	1.12 (1.03 to 1.22)	1.00 (0.89 to 1.12)
**C03CA01**	Furosemide	1317 / 1201	1.10 (1.01 to 1.19)	1.05 (0.95 to 1.17)
**C03DA01**	Spironolactone	258 / 219	1.18 (0.98 to 1.41)	1.17 (0.92 to 1.48)
**C05AA01**	Hydrocortisone	3 / 12	0.25 (0.07 to 0.89)	0.21 (0.05 to 0.83)
**C07AB02**	Metoprolol	2579 / 2289	1.13 (1.07 to 1.19)	1.11 (1.02 to 1.20)
**C07AB03**	Atenolol	252 / 285	0.88 (0.75 to 1.05)	0.79 (0.65 to 0.96)
**C08CA01**	Amlodipine	706 / 718	0.98 (0.88 to 1.09)	0.96 (0.85 to 1.09)
**C08CA02**	Felodipine	112 / 93	1.20 (0.91 to 1.59)	1.34 (0.96 to 1.87)
**C08CA05**	Nifedipine	199 / 226	0.88 (0.73 to 1.07)	0.78 (0.62 to 0.97)
**C09AA05**	Ramipril	709 / 683	1.04 (0.93 to 1.15)	0.94 (0.81 to 1.08)

**C09BA02**	Enalapril and diuretics	81 / 101	0.80 (0.60 to 1.07)	0.75 (0.53 to 1.05)
**C09CA01**	Losartan	292 / 310	0.94 (0.80 to 1.11)	0.89 (0.73 to 1.08)
**C09CA06**	Candesartan	360 / 387	0.93 (0.81 to 1.07)	0.92 (0.77 to 1.09)
**C09DA07**	Telmisartan and diuretics	17 / 7	2.43 (1.01 to 5.86)	2.26 (0.85 to 6.05)
**C10AA01**	Simvastatin	1806 / 1748	1.03 (0.97 to 1.10)	0.90 (0.82 to 0.98)
**C10AA03**	Pravastatin	143 / 184	0.78 (0.62 to 0.97)	0.67 (0.52 to 0.88)
**C10AA05**	Atorvastatin	608 / 580	1.05 (0.94 to 1.17)	0.85 (0.74 to 0.98)
**D07AC01**	Betamethasone	76 / 50	1.52 (1.06 to 2.17)	1.31 (0.88 to 1.94)
**G04BD11**	Fesoterodine	31 / 47	0.66 (0.42 to 1.04)	0.53 (0.27 to 1.04)
**H02AB02**	Dexamethasone	19 / 8	2.38 (1.04 to 5.43)	2.04 (0.77 to 5.35)
**H02AB06**	Prednisolone	963 / 823	1.17 (1.07 to 1.28)	1.05 (0.94 to 1.17)
**H03AA01**	Levothyroxine sodium	629 / 648	0.97 (0.87 to 1.09)	0.92 (0.80 to 1.06)
**J01AA02**	Doxycycline	324 / 213	1.52 (1.28 to 1.81)	1.51 (1.25 to 1.83)
**J01CA04**	Amoxicillin	327 / 168	1.95 (1.62 to 2.34)	2.09 (1.69 to 2.58)
**J01CA08**	Pivmecillinam	309 / 211	1.46 (1.23 to1.74)	1.45 (1.19 to 1.78)
**J01CE02**	Phenoxymethylpenicillin	423 / 277	1.53 (1.31 to 1.77)	1.51 (1.29 to 1.77)
**J01EA01**	Trimethoprim	149 / 114	1.31 (1.02 to 1.67)	1.38 (1.03 to 1.84)
**J01FA01**	Erythromycin	156 / 89	1.75 (1.35 to 2.27)	1.66 (1.22 to 2.24)
**J01FA09**	Clarithromycin	59 / 30	1.97(1.27 to 3.05)	1.59 (1.00 to 2.53)
**J01FF01**	Clindamycin	72 / 49	1.47 (1.02 to 2.11)	1.39 (0.91 to 2.12)
**J01MA02**	Ciproflolaxin	194 / 134	1.45 (1.16 to 1.80)	1.43 (1.10 to 1.86)
**J01XE01**	Nitrofurantoin	105 / 66	1.59 (1.17 to 2.16)	1.62 (1.12 to 2.33)
**L03AA13**	pegfigrastim	16 / 8	2.00 (0.86 to 4.67)	2.41 (0.75 to 7.73)
**L04AB01**	Etanercept	15 / 22	0.68 (0.35 to 1.31)	0.54(0.24 to 1.21)
**M01AB05**	Diclofenac	504 / 387	1.30 (1.14 to 1.49)	1.26(1.07 to 1.47)
**M04AA01**	Allopurinol	351 / 394	0.89 (0.77 to 1.03)	0.85 (0.70 to 1.04)
**N02AA01**	Morphine	137 / 67	2.04 (1.53 to 2.74)	1.84 (1.21 to 2.79)
**N02AA05**	Oxycodone	252 / 191	1.32 (1.09 to 1.59)	1.40 (1.07 to 1.84)
**N02AA59**	Codeine, combinations	1386 / 1202	1.15 (1.07 to 1.25)	1.14 (1.03 to 1.25)
**N02AG01**	Morphine and antispasmodics	28 / 2	14.0 (3.34 to 58.8)	6.66 (2.31 to 19.3)
**N02AX02**	Tramadol	512 / 431	1.19 (1.05 to 1.35)	1.24 (1.05 to 1.45)

**N02BE01**	Paracetemol	1412 / 1290	1.09 (1.01 to 1.18)	1.02 (0.93 to 1.12)
**N03AE01**	Clonazepam	51 / 69	0.74 (0.51 to 1.06)	0.60 (0.36 to 1.01)
**N03AX16**	Pregabalin	112 / 128	0.88 (0.68 to 1.13)	0.69 (0.48 to 0.98)
**N05AD01**	Haloperidol	55 / 35	1.57 (1.03 to 2.40)	1.71 (0.88 to 3.31)
**N05BA01**	Diazepam	664 / 575	1.15 (1.03 to 1.29)	1.06 (0.93 to 1.22)
**N05BA04**	Oxazepam	828 / 757	1.09 (0.99 to 1.21)	1.04 (0.92 to 1.17)
**N05CF01**	Zopiclone	1618 / 1460	1.11 (1.03 to1.19)	1.06 (0.97 to 1.17)
**N05CF02**	Zolpidem	175 / 191	0.92 (0.75 to 1.12)	0.85 (0.67 to 1.07)
**N06AB04**	Citalopram	170 / 182	0.93 (0.76 to 1.15)	0.85 (0.66 to 1.11)
**N06AB06**	Sertraline	90 / 98	0.92 (0.69 to 1.22)	0.78 (0.53 to 1.13)
**N06AB10**	Escitalopram	333 / 352	0.95 (0.81 to 1.10)	0.89 (0.73 to 1.09)
**N06AX16**	Venlafaxine	91 / 103	0.88 (0.67 to 1.17)	0.79 (0.54 to 1.15)
**P01AB01**	Metronidazole	51 / 51	1.00 (0.68 to 1.47)	0.60 (0.38 to 0.94)
**R01AD09**	Mometasone	83 / 100	0.83 (0.62 to 1.11)	0.74 (0.54 to 1.03)
**R03AC02**	Salbutamol	653 / 532	1.23 (1.09 to 1.37)	1.16 (1.00 to 1.34)
**R03AC03**	Terbutaline	92 / 77	1.19 (0.88 to 1.62)	1.24 (0.89 to 1.73)
**R03AK06**	Salmeterol and other anti-asthmatics	250 / 278	0.90 (0.76 to 1.07)	0.83 (0.69 to 1.00)
**R03BA02**	Budesonide	40 / 56	0.71 (0.48 to 1.07)	0.71 (0.44 to 1.14)
**R03BA05**	Fluticasone	34 / 42	0.81 (0.52 to 1.27)	0.66 (0.39 to 1.12)
**R05CB01**	Acetylcysteine	519 / 394	1.32 (1.16 to 1.50)	1.11 (0.96 to1.30)
**R05DA01**	Ethylmorphine	288 / 199	1.45 (1.21 to 1.73)	1.35 (1.10 to 1.66)
**R05FA02**	Opium derivatives and expectorants	71 / 35	2.03 (1.35 to 3.04)	1.59 (1.00 to 2.54)
**S01EA05**	Brimonidine	37 / 10	3.70 (1.84 to 7.44)	3.21 (1.51 to 6.84)
**S01ED02**	Betaxolol	7 / 18	0.39 (0.16 to 0.93)	0.46 (0.18 to 1.18)

[a] Case-crossover analysis, case period (1 to 7 days) and control period (15 to 21 days) before the index-date for the diagnosis of AMI.

[b] Data from the Norwegian Prescription Database, 2004-2014

[c] In BOLASSO, the effect of each selected drugs is controlled for the effect of other selected drugs.

[d] Classified according to Anatomical Therapeutic Chemical (ATC), 5th level

Table S2. Relative risks of acute myocardial infarction within 7 days after the drug was dispensed[a]. Univariable and BOLASSO results for drugs selected by BOLASSO in Sweden[b]

ATC[d]	Drug Name	Case window (*N* exposed) /Control window (*N* exposed)	Univariable	BOLASSO[c]
			RR (95% CI)	RR (95% CI)
**A02AD01**	Ordinary Salt Combinations	58 /34	1.71 (1.12 to 2.60)	1.44 (0.91 to 2.28)
**A02BC01**	Omeprazole	8870 / 8261	1.07 (1.04 to 1.11)	1.12 (1.07 to 1.18)
**A02BC05**	Esomeprazole	871 / 802	1.09 (0.99 to 1.20)	1.09 (0.96 to 1.25)
**A02BX02**	Sucralfate	43 /22	1.95 (1.17 to 3.27)	2.53 (1.21 to 5.29)
**A03AB02**	Glycopyrronium Bromide	52 / 8	6.50 (3.09 to 13.7)	4.19 (1.77 to 9.89)
**A03AD01**	Papaverine	96 / 68	1.41 (1.03 to 1.93)	1.54 (1.05 to 2.25)
**A03FA01**	Metoclopramide	709 / 503	1.41 (1.26 to 1.58)	1.49 (1.28 to 1.73)
**A06AB08**	Sodium picosulfate	632 / 621	1.02(0.91 to 1.14)	0.87 (0.77 to 0.99)
**A06AG10**	Docusate sodium, incl. combinations	53 /22	2.41 (1.47 to 3.96)	1.79 (1.03 to 3.10)
**A07AA02**	Nystatin	372 / 248	1.50 (1.28 to 1.76)	1.48 (1.23 to 1.78)
**A10AD05**	Insulin Aspart	889 / 907	0.98 (0.89 to 1.08)	0.95 (0.86 to 1.04)
**A10AE04**	Insulin Glargine	389 / 462	0.84 (0.74 to 0.96)	0.85 (0.72 to 1.00)
**A10BA02**	Metformin	2659 / 2896	0.92 (0.87 to 0.97)	0.86 (0.79 to 0.94)
**A10BB01**	Glibenclamide	1002 / 1054	0.95 (0.87 to 1.04)	0.91 (0.79 to 1.04)
**A11CC03**	Alfacalcidol	458 /484	0.95 (0.83 to 1.08)	0.86 (0.70 to 1.06)
**A11EX**	Vitamin B-Complex, other combinations	299 / 334	0.90 (0.77 to 1.05)	0.87 (0.73 to1.05)
**A11HA03**	Tocopherol	31 / 46	0.67 (0.43 to 1.06)	0.58 (0.33 to 1.03)
**A12BA01**	Potassium Chloride	2035 / 1914	1.06 (0.99 to 1.13)	1.10 (0.98 to 1.23)
**B01AB05**	Enoxaparin	126 / 93	1.35 (1.04 to 1.77)	1.34 (0.98 to 1.83)
**B01AC04**	Clopidogrel	1290 / 852	1.51 (1.39 to 1.65)	1.78 (1.57 to 2.01)
**B01AC06**	Acetylsalicylic acid	15489 / 15240	1.02 (0.99 to 1.04)	0.99 (0.95 to 1.03)
**B01AC07**	Dipyridamole	288 / 343	0.84 (0.72 to 0.98)	0.87 (0.72 to 1.03)
**B01AC24**	Ticagrelor	96 / 14	6.86 (3.91 to 12.0)	5.14 (2.85 to 9.26)
**B01AF01**	Rivaroxaban	31 / 16	1.94 (1.06 to 3.54)	2.31 (0.96 to 5.55)
**B03AA07**	Ferrous sulfate	2429 / 2320	1.05 (0.99 to 1.11)	1.07 (0.97 to 1.19)
**B03AC**	Iron, parenteral preparations	48 / 29	1.66 (1.04 to 2.62)	1.55 (0.93 to 2.57)

**B03BA01**	Cyanocobalamin	7517 / 7514	1.00 (0.97 to 1.03)	0.99 (0.93 to 1.06)
**B03BA03**	Hydroxocobalamin	125 / 155	0.81 (0.64 to 1.02)	0.79 (0.61 to 1.01)
**B03BB01**	Folic Acid	4398 / 4474	0.98 (0.94 to 1.02)	0.90 (0.82 to 0.98)
**B03XA01**	Erythropoietin	109 / 128	0.85 (0.66 to 1.10)	0.75 (0.56 to 1.00)
**C01AA05**	Digoxin	2425 / 2387	1.02 (0.96 to 1.07)	0.94 (0.85 to 1.04)
**C01DA02**	Glyceral Trinitrate	5020 / 2949	1.70 (1.63 to 1.78)	1.84 (1.74 to 1.94)
**C03AA01**	Bendroflumethiazide	1376 / 1398	0.98 (0.91 to 1.06)	0.95 (0.86 to 1.05)
**C03AB01**	Bendroflumethiazide and potassium	68 / 100	0.68 (0.50 to 0.93)	0.56 (0.38 to 0.83)
**C03CA01**	Furosemide	14307 / 13807	1.04 (1.01 to 1.06)	1.04 (0.99 to 1.09)
**C03DA01**	Spironolactone	2716 / 2674	1.02 (0.96 to 1.07)	0.94 (0.86 to 1.03)
**C03EA01**	Hydrochlorothiazide and potassium-sparing agents	974 / 1082	0.90 (0.83 to 0.98)	0.80 (0.71 to 0.91)
**C07AB02**	Metoprolol	8861 / 8437	1.05 (1.02 to 1.08)	1.05 (1.00 to 1.11)
**C07AB03**	Atenolol	2836 / 2823	1.00 (0.95 to 1.06)	1.01 (0.93 to 1.09)
**C07AB07**	Bisoprolol	2727 / 2562	1.06 (1.01 to 1.12)	1.06 (0.97 to 1.15)
**C08CA01**	Amlodipine	2512 / 2544	0.99 (0.93 to 1.04)	0.92 (0.85 to 1.00)
**C08CA02**	Felodipine	3529 / 3617	0.98 (0.93 to 1.02)	0.91 (0.85 to0.98)
**C09AA02**	Enalapril	5783 / 5716	1.01 (0.98 to 1.05)	0.98 (0.92 to1.04)
**C09AA05**	Ramipril	1870 / 1855	1.01 (0.95 to 1.07)	0.92 (0.83 to1.02)
**C09CA01**	Losartan	1728 / 1738	0.99 (0.93 to 1.06)	0.95 (0.86 to1.05)
**C09CA04**	Irbesartan	233 / 263	0.89 (0.74 to 1.06)	0.78 (0.61 to 1.01)
**C09CA06**	Candesartan	1363 / 1448	0.94 (0.87 to 1.01)	0.88 (0.79 to 0.98)
**C10AA01**	Simvastatin	5611 / 5672	0.99 (0.95 to 1.03)	0.94 (0.88 to 0.99)
**D07AB02**	Hydrocortisone butyrate	143 / 199	0.72 (0.58 to 0.89)	0.78 (0.61 to 0.98)
**G03CA03**	Estradiol	483 / 545	0.89 (0.78 to 1.00)	0.86 (0.75 to 0.99)
**G03XC01**	Raloxifene	28 / 18	1.56 (0.86 to 2.81)	2.36 (0.86 to 6.48)
**G04BD07**	Tolterodine	582 / 629	0.93 (0.83 to 1.04)	0.84 (0.69 to 1.03)
**G04BE03**	Sildenafil	135 / 180	0.75 (0.60 to 0.94)	0.75 (0.58 to 0.97)
**G04BE08**	Tadalafil	97 / 132	0.73 (0.57 to 0.96)	0.73 (0.54 to 0.98)
**G04CA01**	Alfuzosin	824 / 855	0.96 (0.86 to 1.06)	0.87 (0.76 to 1.00)
**G04CB01**	Finasteride	375 / 494	0.76 (0.66 to 0.87)	0.74 (0.64 to 0.86)
**H02AB01**	Betamethasone	802 / 568	1.41 (1.27 to 1.57)	1.21 (1.06 to 1.37)

**H02AB06**	Prednisolone	3692 / 3486	1.06 (1.01 to 1.11)	1.05 (0.97 to 1.13)
**H03AA01**	Levothyroxine Sodium	4843 / 4863	0.99 (0.96 to 1.04)	0.93 (0.86 to 1.01)
**J01AA02**	Doxycycline	1095 / 591	1.85 (1.68 to 2.05)	1.76 (1.58 to 1.96)
**J01CA04**	Amoxicillin	575 / 353	1.63 (1.43 to 1.86)	1.44 (1.24 to 1.66)
**J01CA08**	Pivmecillinam	535 / 422	1.27 (1.12 to 1.44)	1.21 (1.05 to 1.41)
**J01CE02**	Phenoxymethylpenicillin	847 / 595	1.42 (1.28 to 1.58)	1.34 (1.20 to 1.50)
**J01CF05**	Flucloxacillin	872 / 943	0.92 (0.84 to 1.01)	0.89 (0.80 to 0.99)
**J01DB05**	Cefadroxil	226 / 157	1.44 (1.17 to 1.76)	1.39 (1.09 to1.78)
**J01EA01**	Trimethoprim	402 / 281	1.43 (1.23 to 1.67)	1.60 (1.33 to1.92)
**J01EE01**	Sulfamethoxazole and trimethoprim	175 / 119	1.47 (1.17 to 1.86)	1.49 (1.15 to1.93)
**J01FA01**	Erythromycin	90 / 36	2.50 (1.70 to 3.68)	2.14 (1.39 to3.30)
**J01MA02**	Ciproflolaxin	830 / 630	1.32 (1.19 to 1.46)	1.35 (1.19 to1.52)
**J01XE01**	Nitrofurantoin	365 / 249	1.47 (1.25 to 1.72)	1.56 (1.29 to1.89)
**J02AC01**	Fluconazole	232 / 164	1.41 (1.16 to 1.73)	1.34 (1.06 to1.69)
**M01AB05**	Diclofenac	1180 / 1001	1.18 (1.08 to 1.28)	1.15 (1.04 to1.28)
**M01AH05**	Etoricoxib	101 / 118	0.86 (0.66 to 1.12)	0.77 (0.57 to1.04)
**M03BA02**	Carisoprodol	13 / 29	0.45 (0.23 to 0.86)	0.52 (0.26 to1.03)
**M04AA01**	Allopurinol	1897 / 1952	0.97 (0.91 to 1.04)	0.93 (0.83 to1.03)
**M05BA04**	Alendronic Acid	1172 / 1204	0.97 (0.90 to 1.05)	0.92 (0.80 to1.05)
**N02AA01**	Morphine	2195 / 1515	1.45 (1.36 to 1.55)	1.69 (1.52 to1.89)
**N02AA05**	Oxycodone	2659 / 2337	1.14 (1.08 to 1.20)	1.21 (1.10 to1.34)
**N02AA59**	Codeine, combinations	1526 / 1391	1.10 (1.02 to 1.18)	1.09 (1.00 to1.20)
**N02AB03**	Fentanyl	670 / 514	1.30 (1.16 to 1.46)	1.28 (1.11 to1.48)
**N02AC04**	Dextropropoxyphene	1056 / 1050	1.01 (0.92 to 1.10)	0.93 (0.82 to1.05)
**N02AG01**	Morphine and antispasmodics	256 / 42	6.10 (4.40 to 8.45)	5.92(4.09 to8.57)
**N02AG02**	Ketobemidone and antispasmodics	50 / 17	2.94 (1.70 to 5.10)	2.28 (1.18 to4.42)
**N02BE01**	Paracetemol	14481 / 14036	1.03 (1.01 to 1.06)	1.01 (0.97 to1.05)
**N04BA02**	Levodopa and decarboxylase Inhibitor	1243 / 1254	0.99 (0.92 to 1.07)	0.89 (0.75 to1.06)
**N05AX08**	Risperidone	1842 / 1795	1.03 (0.96 to 1.10)	1.14 (0.97 to1.35)
**N05BA01**	Diazepam	1165 / 957	1.22 (1.12 to 1.33)	1.12 (0.99 to1.26)
**N05CD03**	Flunitrazepam	556 / 575	0.97 (0.86 to 1.09)	0.84 (0.70 to1.02)

**N05CD08**	Midazolam	47 / 8	5.88 (2.78 to 12.4)	2.31(1.14 to4.65)
**N05CF01**	Zopiclone	5999 / 6024	1.00 (0.96 to 1.03)	0.96 (0.90 to1.02)
**N05CF02**	Zolpidem	2555 / 2564	1.00 (0.94 to 1.05)	0.97 (0.89 to1.05)
**N06AB04**	Citalopram	5548 / 5513	1.01 (0.97 to 1.04)	0.97 (0.90 to1.05)
**N06AB06**	Sertraline	1358 / 1360	1.00 (0.93 to 1.08)	0.90 (0.78 to1.04)
**N06AX11**	Mirtazapine	2809 / 2754	1.02 (0.97 to1.08)	1.06 (0.95 to1.18)
**P01AB01**	Metronidazole	169 / 185	0.91 (0.74 to1.13)	0.73 (0.57 to0.93)
**R01AD09**	Mometasone	326 / 338	0.96 (0.83 to1.12)	0.87 (0.73 to1.03)
**R03AC02**	Salbutamol	618 / 526	1.17 (1.05 to1.32)	1.12 (0.98 to1.28)
**R03AC03**	Terbutali	968 / 787	1.23 (1.12 to1.35)	1.16 (1.04 to1.30)
**R03AC12**	Salmeterol	73 / 56	1.30 (0.92 to1.85)	1.35 (0.92 to1.98)
**R03AK07**	Formoterol and budesonide	941 / 810	1.16 (1.06 to1.28)	1.12 (1.00 to1.25)
**R03AL02**	Salbutamol and ipratropium bromide	387 / 322	1.20 (1.04 to1.39)	1.13 (0.95 to1.34)
**R03BB04**	Tiotropium Bromide	857 / 823	1.04 (0.95 to1.15)	0.94 (0.84 to1.06)
**R03DA02**	Choline theophyllinate	105 / 59	1.78 (1.29 to2.45)	1.69 (1.12 to2.55)
**R05CB01**	Acetylcysteine	1677 / 1375	1.22 (1.14 to1.31)	1.14 (1.05 to1.24)
**R05CB10**	Mucolytics	733 / 422	1.74 (1.54 to1.96)	1.53 (1.34 to1.75)
**R05DA20**	Opium Alkaloids and derivatives	62 / 23	2.70 (1.67 to4.35)	2.37 (1.35 to4.16)
**R05FA02**	Opium Derivatives and expectorants	828 / 438	1.89 (1.68 to2.12)	1.73 (1.52 to1.97)
**S01BA02**	Hydrocortisone	17 / 40	0.43 (0.24 to0.75)	0.53 (0.28 to1.02)
**S01ED51**	Timolol, Combinations	508 / 481	1.06 (0.93 to1.20)	1.10 (0.95 to1.27)
**V03AE02**	Sevelamer	95 / 77	1.23 (0.91 to1.67)	1.39 (0.99 to1.97)
**Y92BA00**	Teststickor För Blodglukos	1634 / 1667	0.98 (0.92 to1.05)	0.95 (0.87 to1.04)
**Y92EA00**	Lancetter Till Blodprovstagare	325 / 286	1.14 (0.97 to1.33)	1.18 (0.98 to1.43)

[a] Case-crossover analysis, case period (1 to 7 days) and control period (15 to 21 days) before the index-date for the diagnosis of AMI.

[b] Data from the Swedish Prescribed Drug Register, 2005 to 2014

[c] In BOLASSO, the effect of each selected drugs is controlled for the effect of other selected drugs.

[d] Classified according to Anatomical Therapeutic Chemical (ATC), 5th level

Table S3 Relative risks of acute myocardial infarction within 14 days after the drug was dispensed[a], selected by BOLASSO[b] approach in both countries.

		SWEDEN[c]			NORWAY[d]			TOTAL[e]
**ATC[f]**	**Drug Names**	**Case window (exposed); N**	**Control window (exposed); N**	**RR (95% CI)**	**Case window (exposed); N**	**Control window (exposed); N**	**RR (95% CI)**	**Combined Estimate RR (95% CI)**
**A02BC01**	Omeprazole	16268	14553	1.19 (1.14 -1.24)	411	369	1.12 (0.93-1.34)	1.18 (1.13-1.23)
**A02BC05**	Esomeprazole	1561	1364	1.22 (1.09-1.37)	1261	1025	1.29 (1.16-1.43)	1.26 (1.16-1.36)
**A03FA01**	Metoclopramide	1181	790	1.48 (1.31-1.68)	529	307	1.60 (1.32-1.93)	1.52 (1.37-1.68)
**A07AA02**	Nystatin	633	362	1.55 (1.35 -1.79)	113	79	1.39 (0.99-1.96)	1.53 (1.34 -1.74)
**A10BA02**	Metformin	5076	5268	0.87 (0.82-0.93)	1297	1301	0.94 (0.84-1.04)	0.89 (0.84 -0.94)
**A12AX**	Calcium	6480	6440	0.97 (0.91-1.03)	953	971	0.90 (0.78-1.03)	0.96 (0.91-1.01)
**B01AA03**	Warfarin	2285	2357	0.90 (0.85-0.96)	920	940	0.91 (0.81-1.01)	0.90 (0.86-0.95)
**B01AB05**	Enoxaparin	219	153	1.44 (1.11-1.87)	134	88	1.65 (1.15-2.37)	1.51 (1.22-1.86)
**B01AC04**	Clopidogrel	2157	1565	1.58 (1.44-1.74)	578	395	1.53 (1.29-1.80)	1.57 (1.44-1.70)
**B01AC06**	Acetylsalicylic Acid	29062	27782	0.98 (0.95-1.01)	5678	5178	1.03 (0.98-1.09)	0.99 (0.97-1.02)
**B01AC24**	Ticagrelor	108	19	4.20 (2.42-7.30)	39	7	5.21 (2.18-12.5)	4.47 (2.80-7.12)
**C01DA02**	Glyceral Trinitrate	8094	4635	1.87 (1.79-1.96)	2668	1278	2.29 (2.11-2.48)	1.96 (1.89-2.04)
**C01DA14**	Isosorbide Mononitrate	10659	9412	1.08 (1.02-1.13)	1935	1659	1.06 (0.96-1.17)	1.08 (1.03-1.13)
**C03AB01**	Bendroflumethiazide & Potassium	141	186	0.68 (0.51-0.91)	229	166	1.48 (1.17-1.88)	1.08 (0.90- 1.30)
**C03CA01**	Furosemide	26628	24790	1.08 (1.03-1.12)	2395	2226	1.01 (0.93 -1.11)	1.07 (1.03 -1.11)
**C03DA01**	Spironolactone	5025	4805	0.96 (0.89-1.03)	418	419	0.87 (0.72-1.05)	0.95 (0.89-1.02)
**C07AB02**	Metoprolol	16589	15251	1.08 (1.04-1.13)	4635	4118	1.09 (1.02-1.16)	1.08 (1.05-1.12)
**C07AB07**	Bisoprolol	5082	4588	1.09 (1.01-1.16)	440	388	1.15 (0.95-1.39)	1.10 (1.03-1.17)
**C08CA01**	Amlodipine	4814	4774	0.93 (0.88-0.99)	1308	1345	0.88 (0.80-0.97)	0.92 (0.87-0.96)
**C09AA03**	Isinopril	384	399	0.85 (0.68-1.06)	371	317	1.18 (0.98-1.42)	1.03 (0.89-1.19)
**C09CA03**	Valsartan	347	376	0.76 (0.62-0.94)	219	184	1.17 (0.93-1.47)	0.92 (0.79-1.08)
**C09CA06**	Candesartan	2639	2618	0.96 (0.89-1.04)	693	669	1.15 (0.98-1.37)	0.99 (0.92-1.06)
**C10AA01**	Simvastatin	10637	10262	0.94 (0.90-0.98)	3322	3210	0.92 (0.86-0.98)	0.93 (0.90-0.97)
**C10AA03**	Pravastatin	311	364	0.74 (0.60-0.91)	280	296	0.77 (0.62-0.96)	0.75 (0.65-0.88)

**C10AA05**	Atorvastatin	1784	1639	0.97 (0.88-1.06)	1130	1003	0.94 (0.84-1.05)	0.96 (0.89-1.03)
**G03FA01**	Norethisterone And Estrogen	87	120	0.68 (0.48-0.96)	51	70	0.73 (0.50-1.07)	0.70 (0.54-0.91)
**G04BD07**	Tolterodine	1107	1165	0.77 (0.66-0.91)	186	205	0.78 (0.58-1.04)	0.77 (0.67-0.89)
**H02AB06**	Prednisolone	6819	6259	1.09 (1.02-1.16)	1690	1419	1.14 (1.04-1.25)	1.11 (1.05-1.17)
**H03AA01**	Levothyroxine Sodium	9399	9173	0.96 (0.90-1.03)	1212	1296	0.84 (0.75-0.94)	0.93 (0.88-0.98)
**J01AA02**	Doxycycline	1709	1019	1.54 (1.42-1.68)	524	277	1.72 (1.48-2.01)	1.58 (1.47-1.70)
**J01CA04**	Amoxicillin	937	599	1.48 (1.32-1.66)	511	296	1.59 (1.34-1.89)	1.51 (1.38-1.66)
**J01CA08**	Pivmecillinam	934	695	1.32 (1.19-1.48)	507	409	1.26 (1.09-1.47)	1.30 (1.19-1.42)
**J01CE02**	Phenoxymethylpenicillin	1332	985	1.26 (1.15-1.38)	675	452	1.46 (1.28-1.66)	1.32 (1.23-1.43)
**J01FA01**	Erythromycin	130	78	1.68 (1.21-2.34)	232	142	1.52 (1.21-1.91)	1.57 (1.30-1.89)
**J01FF01**	Clindamycin	593	516	1.12 (0.97-1.29)	123	72	1.61 (1.15-2.26)	1.18 (1.04-1.35)
**J01MA02**	Ciproflolaxin	1454	970	1.46 (1.33-1.60)	315	238	1.32 (1.09-1.61)	1.43 (1.32-1.56)
**J01XE01**	Nitrofurantoin	610	405	1.64 (1.41-1.90)	171	132	1.40 (1.06-1.86)	1.58 (1.39-1.81)
**L01BC06**	Capecitabine	51	21	2.02 (0.92-4.44)	19	4	2.74 (0.92-8.13)	2.24 (1.19-4.24)
**M01AB05**	Diclofenac	2158	1934	1.08 (1.00-1.16)	848	676	1.22 (1.09-1.36)	1.12 (1.06-1.19)
**M01AX05**	Glucosamine	504	542	0.87 (0.74-1.02)	98	144	0.64 (0.47-0.87)	0.82 (0.71-0.94)
**N02AA01**	Morphine	3629	2468	2.02 (1.83-2.24)	213	119	1.55 (1.10-2.18)	1.98 (1.80-2.18)
**N02AA05**	Oxycodone	4805	3879	1.41 (1.30-1.53)	471	342	1.69 (1.34-2.13)	1.44 (1.33-1.55)
**N02AA59**	Codeine, Combinations	2749	2484	1.12 (1.03-1.21)	2450	2102	1.18 (1.09-1.28)	1.15 (1.09-1.22)
**N02AB03**	Fentanyl	1190	897	1.41 (1.24-1.59)	220	151	1.49 (1.09-2.05)	1.42 (1.27-1.60)
**N02AG01**	Morphine and antispasmodics	314	57	5.86 (4.15-8.27)	32	0	18.74(8.71-40.34)	7.13 (5.20-9.76)
**N02AG02**	Ketobemidone and antispasmodics	74	34	2.45 (1.42-4.21)	30	16	2.40 (0.87-6.61)	2.44 (1.51-3.94)
**N02AX02**	Tramadol	3963	3748	1.06 (0.99-1.13)	905	745	1.22 (1.07-1.38)	1.09 (1.03-1.16)
**N02BE01**	Paracetemol	27229	25369	1.03 (1.00-1.07)	2585	2308	1.06 (0.98-1.14)	1.04 (1.00-1.07)
**N05BA01**	Diazepam	2062	1731	1.19 (1.07-1.31)	1182	1049	1.06 (0.96-1.18)	1.12 (1.05-1.21)
**N05BA04**	Oxazepam	9286	8644	1.09 (1.03-1.15)	1540	1360	1.12 (1.01-1.24)	1.11 (1.03-1.19)
**N05CF01**	Zopiclone	11300	10828	1.03 (0.97-1.08)	2920	2816	0.97 (0.91-1.04)	1.01 (0.97-1.05)
**N06AX11**	Mirtazapine	5299	4958	1.19 (1.08-1.30)	339	302	1.20 (0.94-1.53)	1.19 (1.09-1.30)
**R01AD09**	Mometasone	628	620	0.93 (0.82-1.05)	151	193	0.68 (0.53-0.86)	0.87 (0.78-0.97)
**R03AC02**	Salbutamol	1121	942	1.19 (1.06-1.33)	1176	987	1.20 (1.07-1.34)	1.20 (1.10-1.29)

**R03AK06**	Salmeterol and fluticasone	603	600	0.91 (0.80-1.04)	475	525	0.80 (0.69-0.94)	0.86 (0.78-0.95)
**R03BA02**	Budesonide	1145	1112	0.90 (0.81-0.99)	86	93	0.76 (0.54-1.06)	0.89 (0.81-0.98)
**R03BB04**	Tiotropium Bromide	1627	1661	0.89 (0.81-0.98)	394	417	0.87 (0.74-1.01)	0.88 (0.82-0.96)
**S03CA04**	Hydrocortisone & antiinfectives	456	498	0.90 (0.78-1.02)	58	73	0.75 (0.52-1.08)	0.88 (0.78-1.00)

[a] Case-crossover analysis, case period (1 to 14 days) and control period (29 to 42 days) before the index date for the diagnosis of AMI.[b] In BOLASSO, the effect of each selected drugs is controlled for the effect of other selected drugs.

[c] Data from the Swedish Prescribed Drug Register , 2005 to 2014

[d] Data from the Norwegian Prescription Database, 2004 to 2014

[e] Combined estimates of Norwegian and Swedish data calculated using fixed-effect

[f] Classified according to Anatomical Therapeutic Chemical (ATC), 5th level

Table S4 Relative risks of acute myocardial infarction within 7 days after the drug was dispensed[a], selected by BOLASSO[b] approach in both countries. AMI diagnosed in hospital only.

		SWEDEN[c]			NORWAY[d]			TOTAL[e]
ATC[f]	Drug Names	Case window (exposed); N	Control window (exposed); N	RR (95% CI)	Case window (exposed); N	Control window (exposed); N	RR (95% CI)	Combined Estimate RR (95% CI)
	Narcotic analgesics							
**R05FA02**	Opium derivatives and expectorants	695	391	1.60 (1.39-1.85)	61	29	1.66 (1.00-2.75)	1.60 (1.40- 1.84)
**N02AA05**	Oxycodone	1952	1735	1.17 (1.04-1.31)	195	146	1.45 (1.09-1.94)	1.21 (1.08- 1.34)
**N02AA59**	Codeine, combinations	1234	1143	1.06 (0.96-1.17)	1078	991	1.07 (0.97-1.18)	1.07 (0.99-1.14)
	Antibiotics							
**J01FA01**	Erythromycin	82	30	2.37 (1.47-3.84)	130	71	1.77 (1.27-2.48)	1.95 (1.48- 2.56)
**J01XE01**	Nitrofurantoin	316	189	1.74 (1.42-2.13)	92	54	1.71 (1.14-2.58)	1.73 (1.45- 2.08)
**J01EA01**	Trimethoprim	318	202	1.79 (1.45-2.20)	119	90	1.42 (1.03 1.95)	1.67 (1.40- 1.99)
**J01AA02**	Doxycycline	916	526	1.63 (1.45-1.84)	264	174	1.52 (1.23-1.89)	1.60 (1.45- 1.78)
**J01CA04**	Amoxicillin	456	295	1.38 (1.17-1.62)	264	135	2.12 (1.69-2.67)	1.59 (1.40-1.82)
**J01MA02**	Ciproflolaxin	677	504	1.38 (1.20-1.58)	156	103	1.55 (1.16-2.07)	1.41 (1.25- 1.60)
**J01EE01**	Sulfamethoxazole & trimethoprim	142	100	1.39 (1.06-1.84)	70	43	1.46 (0.96-2.23)	1.41 (1.12- 1.78)
**J01CE02**	Phenoxymethylpenicillin	702	514	1.30 (1.14-1.47)	337	226	1.45 (1.21-1.75)	1.35 (1.21- 1.50)
**J01CA08**	Pivmecillinam	442	351	1.23 (1.05-1.45)	256	173	1.50 (1.21-1.85)	1.32 (1.16- 1.50)
	Antithrombotic agents							
**B01AC24**	Ticagrelor	94	13	5.22 (2.83-9.64)	26	6	3.11 (1.26-7.70)	4.44 (2.67- 7.37)
**B01AC04**	Clopidogrel	1126	701	1.82 (1.61-2.06)	319	214	1.40 (1.14-1.72)	1.70 (1.53-1.89)
**B01AB05**	Enoxaparin	107	72	1.41 (1.00-2.00)	66	29	2.46 (1.35-4.48)	1.62 (1.20-2.19)
**B01AC06**	Acetylsalicylic acid	11864	11515	1.01 (0.96-1.05)	2694	2396	1.11 (1.02-1.20)	1.03 (0.99-1.07)
	Adrenergics							
**R03AC03**	Terbutaline	831	667	1.16 (1.03-1.31)	75	58	1.42 (0.96-2.10)	1.18 (1.05-1.32)
**R03AC02**	Salbutamol	527	449	1.12 (0.97-1.30)	510	426	1.12 (0.95-1.32)	1.12 (1.00- 1.25)
	Protontopump inhibitor							
**A02BC01**	Omeprazole	6698	6126	1.14 (1.07-1.20)	204	186	1.17 (0.92-1.48)	1.15 (1.08- 1.21)
	Vasodilator							
**C01DA02**	Glyceral trinitrate	4607	2656	1.88 (1.78-1.99)	1549	729	2.27 (2.05-2.52)	1.96 (1.87-2.06)
	Antiinflammatory drug							
**M01AB05**	Diclofenac	1033	854	1.19 (1.07-1.33)	434	340	1.19 (1.02-1.40)	1.19 (1.09-1.30)
	Prokinetic/antiemetic drug							

**A03FA01**	Metoclopramide	464	348	1.40 (1.16-1.69)	232	153	1.39 (1.06-1.81)	1.40 (1.20-1.63)
	Mucolytics							
**R05CB01**	Acetylcysteine	1360	1105	1.15 (1.05-1.27)	394	312	1.07 (0.91-1.26)	1.13 (1.04-1.23)
	Glucocorticoid							
**H02AB06**	Prednisolone	2883	2659	1.06 (0.98-1.15)	804	671	1.08 (0.95-1.23)	1.07 (1.00-1.14)
	Betatoblocker							
**C07AB02**	Metoprolol	6969	6602	1.03 (0.98-1.09)	2152	1909	1.09 (1.00-1.20)	1.05 (1.00-1.09)
	Nontonarcotic analgesic							
**N02BE01**	Paracetemol	10453	10145	1.02 (0.98-1.07)	1165	1059	1.04 (0.93-1.15)	1.02 (0.98-1.06)
	Anxiolytic							
**N05BA04**	Oxazepam	3331	3250	0.99 (0.92-1.07	656	597	1.04 (0.91-1.19)	1.00 (0.94-1.07)
	Hypnotics and sedatives							
**N05CF01**	Zopiclone	4299	4312	0.95 (0.89-1.01)	1302	1187	1.05 (0.95-1.16)	0.98 (0.93-1 .03)
**N05CF02**	Zolpidem	2022	2054	0.94 (0.86-1.02)	147	162	0.82 (0.63-1.07)	0.93 (0.86-1.01)
	Calcium Channel blockers							
**C08CA02**	Felodipine	2955	2984	0.93 (0.86-1.01)	103	81	1.51 (1.04-2.19)	0.95 (0.88- 1.03)
**C08CA01**	Amlodipine	2090	2119	0.91 (0.84-1.00)	600	620	0.95 (0.83-1.09)	0.92 (0.86-0.99)
	ACE inhibitor							
**C09AA05**	Ramipril	1439	1407	0.93 (0.84-1.04)	547	540	0.93 (0.80-1.08)	0.93 (0.85-1.02)
	Lipid modifying agent							
**C10AA01**	Simvastatin	4730	4717	0.95 (0.90-1.01)	1544	1504	0.89 (0.80-0.99)	0.94 (0.89-0.99)
	Antitothyroid agent							
**H03AA01**	Levothyroxine sodium	3530	3597	0.90 (0.83-0.99)	534	556	0.92 (0.80-1.07)	0.91 (0.84-0.98)
	Antidiabetic drug							
**A10BA02**	Metformin	2178	2362	0.86 (0.78-0.94)	584	588	0.95 (0.82-1.10)	0.89 (0.82-0.96)
	Antidepressant							
**N06AB06**	Sertraline	928	949	0.85 (0.73-1.00)	72	80	0.71 (0.47-1.09)	0.83 (0.72-0.96)
	Antiprotozoal drug							
**P01AB01**	Metronidazole	132	141	0.77 (0.58-1.02)	39	43	0.55 (0.32-0.92)	0.72 (0.56-0.92)

[a] Case-crossover analysis, case period (1 to 7 days) and control period (15 to 21 days) before the index-date for the diagnosis of AMI.

[b] In BOLASSO, the effect of each selected drugs is controlled for the effect of other selected drugs.[c] Data from the Swedish Prescribed register, 2005 to 2014

[d] Data from the Norwegian Prescribed Database, 2004 to 2014

[e] Combined estimates of Norwegian and Swedish data calculated using fixed-effect model

[f] Classified according to Anatomical Therapeutic Chemical (ATC), 5th level

Table S5 Relative risks of acute myocardial infarction within 7 days after the drug was dispensed. Univariable results for drugs selected by BOLASSO approach in both the countries. Sub-group analyses according to age in Norway

				Norway‡ < 80 years			Norway‡ ≥80 years
**ATC Code^S**	**Drug Names**	**Case window (exposed); N**	**Control window (exposed); N**	**RR (95% CI)**	**Case window (exposed); N**	**Control window (exposed); N**	**RR (95% CI)**
	Narcotic analgesics						
**N02AG01**	Morphine & antispasmodics	NA	NA	NA	28	2	14.0 (3.33-58.77)
**N02AA01**	Morphine	25	24	1.04 (0.59-1.82)	112	43	2.60 (1.83-3.70)
**N02AA05**	Oxycodone	135	95	1.42 (1.09-1.85)	117	96	1.22 (0.93-1.60)
**N02AA59**	Codeine, combinations	759	686	1.11 (1.00-1.23)	627	516	1.22 (1.08-1.37)
**R05FA02**	Opium derivatives & expectorant	34	22	1.55 (0.90-2.64)	37	13	2.85 (1.51-5.35)
	Non-narcotic analgesic						
**N02BE01**	Paracetemol	517	489	1.05 (0.93-1.19)	895	801	1.12 (1.01-1.23)
	Anti-inflammatory drugs						
**M01AB05**	Diclofenac	344	299	1.15 (0.99-1.34)	160	88	1.82 (1.40-2.36)
	Antibiotics						
**J01FA01**	Erythromycin	95	45	2.11 (1.48-3.01)	61	44	1.39 (0.94-2.04)
**J01AA02**	Doxycycline	176	131	1.34 (1.07-1.68)	148	82	1.80 (1.38-2.36)
**J01CA04**	Amoxicillin	158	84	1.88 (1.44-2.45)	169	84	2.01 (1.55-2.61)
**J01XE01**	Nitrofurantoin	37	19	1.95 (1.12-3.39)	68	47	1.45 (1.00-2.10)
**J01EA01**	Trimethoprim	48	38	1.26 (0.83-1.93)	101	76	1.33 (0.99-1.79)
**J01CE02**	Phenoxymethylpenicillin	215	150	1.43 (1.16-1.77)	208	127	1.64 (1.31-2.04)
**J01MA02**	Ciproflolaxin	86	56	1.54 (1.10-2.15)	108	78	1.38 (1.03-1.85)
**J01CA08**	Pivmecillinam	101	72	1.40 (1.04-1.90)	208	139	1.50 (1.21-1.85)
	Antiprotozoal drug						
**P01AB01**	Metronidazole	26	29	0.90 (0.53-1.52)	25	22	1.14 (0.64-2.02)
	Antithrombotic agents						
**B01AC24**	Ticagrelor	24	5	4.80 (1.83-12.58)	3	1	3.00 (0.31-28.84)
**B01AC04**	Clopidogrel	216	126	1.71 (1.38-2.14)	175	125	1.40 (1.11-1.76)
**B01AB05**	Enoxaparin	41	15	2.73 (1.51-4.94)	37	28	1.32 (0.81-2.16)
**B01AC06**	Acetylsalicylic acid	1448	1185	1.22 (1.13-1.32)	1693	1618	1.05 (0.98-1.12)
	Antiadrenergic agents						
**R03AC03**	Terbutaline	57	43	1.33 (0.89-1.97)	35	34	1.03 (0.64-1.65)
**R03AC02**	Salbutamol	343	281	1.22 (1.04-1.43)	310	251	1.24 (1.05-1.46)

	Proton pump inhibitors						
**A02BC05**	Esomeprazol	305	307	0.99 (0.85-1.16)	420	281	1.49 (1.29-1.74)
**A02BC01**	Omeprazole	108	98	1.10 (0.84-1.45)	130	115	1.13 (0.88-1.45)
	Vasodilator						
**C01DA02**	Glyceral trinitrate	992	390	2.54 (2.26-2.86)	766	474	1.62 (1.44-1.81)
	Prokinetic drug						
**A03FA01**	Metoclopramide	132	98	1.35 (1.04-1.75)	203	122	1.66 (1.33-2.08)
	Expectorants						
**R05CB01**	Acetylcysteine	204	175	1.17 (0.95-1.43)	315	219	1.44 (1.21-1.71)
	Anxiolytics						
**N05BA01**	Diazepam	334	325	1.03 (0.88-1.20)	330	250	1.32 (1.12-1.56)
	Angiotension II antagonists						
**C09CA01**	Losartan	147	139	1.06 (0.84-1.33)	145	171	0.85 (0.68-1.06)
**C09CA06**	Candesartan	180	193	0.93 (0.76-1.14)	180	194	0.93 (0.76-1.14)
	Antidepressants						
**N06AB04**	Citalopram	63	56	1.13 (0.78-1.61)	107	126	0.85 (0.66-1.10)
**N06AB06**	Sertraline	30	34	0.68 (0.43-1.08)	60	54	1.11 (0.77-1.60)
	ACE inhibitors						
**C09AA05**	Ramipril	275	237	1.16 (0.98-1.38)	434	446	0.97 (0.85-1.11)
	Antigout agent						
**M04AA01**	Allopurinol	109	137	0.80 (0.62-1.02)	242	257	0.94 (0.79-1.12)
	Antidiabetic agent						
**A10BA02**	Metformin	361	367	0.98 (0.85-1.14)	332	328	1.01 (0.87-1.18)
	Beta-blocking agents						
**C07AB02**	Metoprolol	1101	925	1.19 (1.09-1.30)	1478	1364	1.09 (1.01-1.17)
**C07AB03**	Atenolol	96	111	0.86 (0.66-1.14)	156	174	0.90 (0.72-1.11)
	Calcium-channel blockers						
**C08CA01**	Amlodipine	329	331	0.99 (0.85-1.16)	377	387	0.97 (0.85-1.12)
**C08CA02**	Felodipine	58	38	1.53 (1.01-2.29)	54	55	0.98 (0.67-1.43)
	Decongestants						
**R01AD09**	Mometasone	62	72	0.86 (0.61-1.21)	21	28	0.75 (0.43-1.32)
	Diuretics						
**C03CA01**	Furosemide	317	301	1.05 (0.90-1.23)	1000	900	1.11 (1.02-1.22)
**C03DA01**	Spironolactone	72	66	1.09 (0.78-1.52)	186	153	1.22 (0.98-1.51)
	Glucocorticoids						
**H02AB06**	Prednisolone	438	362	1.21 (1.05-1.39)	525	461	1.14 (1.00-1.29)

	Hypnotics and sedatives						
**N05CF01**	Zopiclone	608	568	1.07 (0.95-1.20)	1010	892	1.13 (1.03-1.24)
**N05CF02**	Zolpidem	76	77	0.99 (0.72-1.36)	608	568	1.07 (0.95-1.20)
	Lipid modifying agent						
**C10AA01**	Simvastatin	903	846	1.07 (0.97-1.17)	903	902	1.00 (0.91-1.10)
	Antithyroid agent						
**H03AA01**	Levothyroxine sodium	230	257	0.89 (0.75-1.07)	399	391	1.02 (0.89-1.17)
	Antianemic agent						
**B03BA03**	Hydroxocobalamin	20	18	1.11 (0.59-2.10)	29	45	0.64 (0.40-1.03)

NA= Not Available

* Case crossover analysis, case period (1 to 7 days) and control period (15 to 21 days) before the index-date for the diagnosis of first AMI.

† In BOLASSO, the effect of each selected drugs is controlled for the effect of other selected drugs.

‡ Data from the Norwegian Prescribed Database, 2004 to 2014

S Classified according to Anatomical Therapeutic Chemical (ATC), 5th level

**Table S6 Relative risks of acute myocardial infarction within 7 days after the drug was dispensed. Univariable results for drugs selected by BOLASSO approach in both the countries. Sub-group analyses according to age in Sweden**							
				**Sweden‡**			**Sweden‡**

				<80 years			≥80 years
**ATC Code[S]**	**Drug Names**	**Case window (exposed); N**	**Control window (exposed); N**	**RR (95% CI)**	**Case window (exposed); N**	**Control window (exposed); N**	**RR (95% CI)**
	Narcotic analgesics						
**N02AG01**	Morphine & antispasmodics	71	23	3.09 (1.93-4.94)	185	19	9.73 (6.07-15.61)
**N02AA01**	Morphine	665	537	1.23 (1.11-1.39)	1530	978	1.56 (1.44-1.70)
**N02AA05**	Oxycodone	1024	891	1.15 (1.05-1.26)	1635	1446	1.13 (1.05-1.23)
**N02AA59**	Codeine, combinations	929	873	1.06 (0.97-1.16)	597	518	1.16 (1.02-1.30)
**R05FA02**	Opium derivatives & expectorant	527	313	1.68 (1.46-1.94)	301	125	2.40 (1.95-2.97)
	Non-narcotic analgesic						
**N02BE01**	Paracetemol	4336	4304	1.01 (0.97-1.05)	10145	9732	1.04 (1.01-1.07)
	Anti-inflammatory drugs						
**M01AB05**	Diclofenac	838	968	1.20 (1.09-1.33)	342	303	1.12 (0.97-1.32)
	Antibiotics						
**J01FA01**	Erythromycin	66	24	2.75 (1.72-4.39)	24	12	2.00 (1.00-3.99)
**J01AA02**	Doxycycline	648	390	1.66 (1.47-1.88)	447	201	2.22 (1.88-2.63)
**J01CA04**	Amoxicillin	325	226	1.44 (1.21-1.70)	250	127	1.97 (1.59-2.44)
**J01XE01**	Nitrofurantoin	131	109	1.20 (0.93-1.55)	234	140	1.67 (1.36-2.06)
**J01EA01**	Trimethoprim	122	85	1.44 (1.09-1.89)	280	196	1.43 (1.19-1.71)
**J01CE02**	Phenoxymethylpenicillin	437	369	1.18 (1.03-1.36)	410	226	1.81 (1.54-2.13)
**J01MA02**	Ciproflolaxin	419	319	1.31 (1.14-1.52)	411	311	1.32 (1.14-1.53)
**J01CA08**	Pivmecillinam	159	137	1.16 (0.92-1.46)	376	285	1.31 (1.13-1.54)
	Antiprotozoal drug						
**P01AB01**	Metronidazole	75	93	0.81 (0.59-1.09)	94	92	1.02 (0.77-1.36)
	Antithrombotic agents						
**B01AC24**	Ticagrelor	81	11	7.36 (3.92-13.82)	15	3	5.00 (1.44-17.27)
**B01AC04**	Clopidogrel	704	391	1.80 (1.59-2.04)	586	461	1.27 (1.13-1.44)
**B01AB05**	Enoxaparin	58	53	1.09 (0.75-1.59)	68	40	1.70 (1.15-2.51)

**B01AC06**	Acetylsalicylic acid	4956	4746	1.04 (1.00-1.09)	10,533	10,494	1.00 (0.98-1.03)
	Antiadrenergic agents						
**R03AC03**	Terbutaline	645	533	1.21 (1.08-1.36)	323	254	1.27 (1.08-1.50)
**R03AC02**	Salbutamol	418	346	1.21 (1.05-1.39)	200	180	1.11 (0.91-1.36)
	Proton pump inhibitors						
**A02BC05**	Esomeprazol	472	430	1.10 (0.96-1.25)	399	372	1.07 (0.93-1.24)
**A02BC01**	Omeprazole	3328	3012	1.10 (1.05-1.16)	5542	5249	1.06 (1.02-1.10)
	Vasodilator						
**C01DA02**	Glyceral trinitrate	2760	1456	1.90 (1.78-2.02)	2260	1493	1.51 (1.42-1.62)
	Prokinetic drug						
**A03FA01**	Metoclopramide	310	248	1.25 (1.06-1.48)	399	255	1.56 (1.34-1.83)
	Expectorants						
**R05CB01**	Acetylcysteine	828	649	1.28 (1.15-1.41)	849	726	1.17 (1.06-1.29)
	Anxiolytics						
**N05BA01**	Diazepam	669	596	1.12 (1.01-1.25)	496	361	1.37 (1.20-1.57)
	Angiotension II antagonists						
**C09CA01**	Losartan	772	767	1.01 (0.91-1.11)	956	971	0.98 (0.90-1.08)
**C09CA06**	Candesartan	648	684	0.95 (0.85-1.05)	715	764	0.94 (0.85-1.04)
	Antidepressants						
**N06AB04**	Citalopram	1439	1475	0.98 (0.91-1.05)	4109	4038	1.02 (0.97-1.05)
**N06AB06**	Sertraline	498	533	0.93 (0.83-1.06)	860	827	1.04 (0.95-1.14)
	ACE inhibitors						
**C09AA05**	Ramipril	738	726	1.01 (0.92-1.13)	1132	1129	1.00 (0.92-1.09)
	Antigout agent						
**M04AA01**	Allopurinol	597	651	0.92 (0.82-1.02)	1300	1301	0.99 (0.93-1.08)
	Antidiabetic agent						
**A10BA02**	Metformin	1501	1694	0.89 (0.83-1.04)	1158	1202	0.96 (0.89-1.04)
	Beta-blocking agents						

**C07AB02**	Metoprolol	3324	3074	1.08 (1.03-1.14)	5537	5363	1.03 (0.99-1.07)
**C07AB03**	Atenolol	1092	1090	1.00 (0.92-1.09)	1744	1733	1.01 (0.94-1.08)
	Calcium-channel blockers						
**C08CA01**	Amlodipine	1163	1162	1.00 (0.92-1.09)	1349	1382	0.98 (0.91-1.05)
**C08CA02**	Felodipine	1382	1380	1.00 (0.93-1.08)	2147	2237	0.96 (0.90-1.02)
	Decongestants						
**R01AD09**	Mometasone	245	252	0.97 (0.82-1.16)	81	86	0.94 (0.70-1.28)
	Diuretics						
**C03CA01**	Furosemide	3329	3312	1.01 (0.96-1.05)	10978	10495	1.05 (1.02-1.07)
**C03DA01**	Spironolactone	743	761	0.98 (0.88-1.08)	1973	1913	1.03 (0.97-1.10)
	Glucocorticoids						
**H02AB06**	Prednisolone	1330	1184	1.12 (1.04-1.21)	2362	2302	1.03 (0.97-1.09)
	Hypnotics and sedatives						
**N05CF01**	Zopiclone	1898	1938	0.98 (0.92-1.04)	4101	4086	1.00 (0.96-1.05)
**N05CF02**	Zolpidem	932	980	0.95 (0.87-1.04)	1623	1584	1.02 (0.96-1.10)
	Lipid modifying agent						
**C10AA01**	Simvastatin	3239	3233	1.00 (0.95-1.05)	2372	2439	0.97 (0.92-1.03)
	Antithyroid agent						
**H03AA01**	Levothyroxine sodium	1326	1351	0.98 (0.91-1.06)	3517	3512	1.00 (0.96-1.05)
	Antianemic agent						
**B03BA03**	Hydroxocobalamin	34	66	0.52 (0.34-0.78)	91	89	1.02 (0.76-1.37)

* Case crossover analysis, case period (1 to 7 days) and control period (15 to 21 days) before the index-date for the diagnosis of first AMI.
† In BOLASSO, the effect of each selected drugs is controlled for the effect of other selected drugs.
‡ Data from the Swedish Prescribed Drug Register, 2005 to 2014
S Classified according to Anatomical Therapeutic Chemical (ATC), 5th level

Appendix A

The case-crossover design

A.1 The likelihood function under the case-crossover design

In our analysis, the subjects are the patients who experienced the event (case), and each patient is both the case and the control of itself, in different time periods. For the whole analysis, we used one case and one control window for each patient. According to [6], our design is called a non-localizable design and, more specifically, an unidirectional design, because the windows are placed after observing the event for each patient and not a priori.

Assume that we have a total of N individuals who experienced the event at some point in the time set $\{1, ..., T\}$. Here, T is the length of the observation period, and the $t \in \{1, ..., T\}$ are the discrete time points where each case was checked (followed up). Let Y_{it} be the risk occurrence indicator for subject i at time t, that is $Y_{it} = 1$ if the event took place at time t and $Y_{it} = 0$ otherwise. For $i = 1, \ldots, N$ and $t = 1, \ldots, T$, let $X_{it} = (X_{it1}, ..., X_{itp})$ be the p-dimensional vector of exposures at time t for the ith subject. Let further $X_i = (X_{it}; t = 1, ..., T)$ be the corresponding p-dimensional exposure time series vector.

Then, if it is assumed that events follow a proportional hazards model, and if it is assumed that events are rare, we will assume that

$$P\left(Y_{it} = 1 | X_i\right) = \lambda_{0it} e^{X_{it}\beta}, \tag{1}$$

where $\beta = (\beta_1, ...\beta_p)'$ is a vector of coefficients. The baseline function λ_{0it} is assumed to vary among subjects, carrying characteristics of the ith subject like, for example, age, or other characteristics which may change with time t. In other words, Eq. (1) is the probability that the ith subject will experience the event exactly at time t, given its exposure vector and characteristics.

Assume first that the exposure of each subject is observed throughout the time set $\{1, 2, ..., T\}$. Then by conditioning on the occurrence of exactly one event in $\{1, 2, ..., T\}$, and as above assuming that events are rare, the contribution to the likelihood from the ith subject would be ([8, 6]):

$$\begin{aligned} & P\left(Y_{i,t_i} = 1 \middle| \sum_{s=1}^{T} Y_{is} = 1, X_i\right) \\ = & \frac{P\left(Y_{i,t_i} = 1 | X_i\right)}{P\left(\sum_{s=1}^{T} Y_{is} = 1 | X_i\right)} \\ = & \frac{P\left(Y_{i,t_i} = 1 | X_i\right)}{\sum_{s=1}^{T} P\left(Y_{is} = 1 | X_i\right)} \end{aligned}$$

$$= \frac{\lambda_{0it_i} e^{X_{it_i}\beta}}{\sum_{s=1}^{T} \lambda_{0is} e^{X_{is}\beta}} \tag{2}$$

The complete likelihood is then the product of these contributions, namely

$$L(\beta) = \prod_{i=1}^{N} \frac{\lambda_{0it_i} e^{X_{it_i}\beta}}{\sum_{s=1}^{T} \lambda_{0is} e^{X_{is}\beta}}. \tag{3}$$

Since we do not know the exposure X_{it} for all $t \in 1, 2, ..., T$, but only in a referent window W_i, following Lumley and Levy [8], we replace the denominators in Eq. (3) by the simplification where s runs only in the referent window. Thus, we replace Eq. (3) by

$$L(\beta) = \prod_{i=1}^{N} \frac{\lambda_{0it_i} e^{X_{it_i}\beta}}{\sum_{t \in W_i} \lambda_{0it} e^{X_{it}\beta}} = \prod_{i=1}^{N} \frac{e^{X_{it_i}\beta}}{\sum_{t \in W_i} e^{X_{it}\beta}} = \prod_{i=1}^{N} \frac{e^{X_{it_i}\beta}}{e^{X_{it_i}\beta} + e^{X_{is_i}\beta}}. \tag{4}$$

In our application, $W_i = \{t_i, s_i\}$, where t_i and s_i are the case and the control windows of the ith subject, respectively. Furthermore, the λ_{0it_i} and λ_{0it} are cancelled because they are assumed to be (approximately) constant in the referent windows. Now, X_{it_i} is a vector of length p corresponding to the exposure of each drug in the case window, while X_{is_i} is a vector of the same length corresponding to the exposures in the control window. Both vectors contain the values 0 and 1 for non-exposure and exposure, respectively.

It should be noted that Eq. (4) is not an actual likelihood because of the reduction of the sum in the denominator of (3). Thus, as noted by Lumley and Levy [8], the resulting estimates may be biased. This bias, called overlap bias, will however usually be small (see [8]).

On the other hand, Eq. (4) has the form of the likelihood of a conditional logistic regression (CLR) and can hence be analyzed using standard statistical packages allowing CLR, Note that the window W_i in Eq. (4) consists of two time points, t_i (case) and s_i (control), while more controls can be included by adding points in the window.

A.2 Generalized linear model

Our model can be represented differently in order to show that it is a generalized linear model of logistic type. Consider the matrices $X_t = (X_{1t_1}, ..., X_{Nt_N})^T$, corresponding to the case windows, and $X_s = (X_{1s_1}, ..., X_{Ns_N})^T$, corresponding to the control windows, both of dimension $N \times p$, where X_{it_i} and X_{is_i} are defined above. Consider also the corresponding response vectors $Y_t = (Y_{1t_1}, ..., Y_{Nt_N})^T$ and $Y_s = (Y_{1s_1}, ..., Y_{Ns_N})^T$, for the cases and controls, respectively. These are both N-dimensional, where the response vector for the cases contains only the element 1 and the response vector for the controls contains only the element 0. This is because we restrict attention to patients with exactly one event in the

given time set. The likelihood $L(\beta)$ can hence be written in the form

$$L(\beta) = \prod_{i=1}^{N} \frac{e^{X_{it_i}\beta}}{e^{X_{it_i}\beta} + e^{X_{is_i}\beta}} = \prod_{i=1}^{N} \frac{1}{1 + e^{-X_i\beta}}, \tag{5}$$

where $X_i = X_{it_i} - X_{is_i}$, that is, we subtract the control vector from the case vector for each patient. As will be seen below, for a case-crossover design with one case and one control window, the likelihood can be written in the form of the unconditional likelihood for a binary logistic regression with no intercept and constant response equal to one (see Avalos et al.[3]). The fact that the response is constant and equal to one, becomes more clear when we subtract the response vector Y_s from Y_t. Note that now the data matrix X consists of values $-1, 0, 1$. Furthermore, for a specific component β_j of the vector β, e^{β_j} is the relative risk for the event, if the jth component of X_i is increased by 1, which means that the corresponding medicine is taken in the case window and not in the control window.

By letting p_i be the contribution of the ith individual to the likelihood in Eq. (5), that is $p_i = \frac{1}{1+e^{-X_i\beta}} = \frac{e^{X_i\beta}}{1+e^{X_i\beta}}$, then the likelihood can be written in the following form:

$$L(\beta) = \prod_{i=1}^{N} \frac{1}{1 + e^{-X_i\beta}} = \prod_{i=1}^{N} p_i = \prod_{i=1}^{N} p_i^{y_i} (1 - p_i)^{1-y_i}, \tag{6}$$

since $y_i = 1$ for all i. The log-likelihood is of the form

$$\begin{aligned} \ell(\beta) &= \log(L(\beta)) = \sum_{i=1}^{N} \log\left[p_i^{y_i}(1-p_i)^{1-y_i}\right] \\ &= \sum_{i=1}^{N} \left[y_i \log(p_i) + (1-y_i)\log(1-p_i)\right] \\ &= \sum_{i=1}^{N} \left[y_i \text{logit}(p_i) + \log(1-p_i)\right], \end{aligned} \tag{7}$$

which corresponds to the likelihood of logistic regression ([9, 10])

Finally, the log-likelihood function given in Eq. (7) is a concave function with respect to the β coefficients. This can easily be seen by rewriting the function in the following form,

$$\begin{aligned} \ell(\beta) &= \sum_{i=1}^{N} \left[y_i \text{logit}(p_i) + \log(1-p_i)\right] \\ &= \sum_{i=1}^{N} \left(y_i X_i \beta + \log\left(\frac{1}{1+e^{X_i\beta}}\right)\right) \\ &= \sum_{i=1}^{N} \left(y_i X_i \beta - \log\left(1 + e^{X_i\beta}\right)\right) \end{aligned}$$

and noting that $-\log(1 + e^x)$ is a concave function.

A.3 The lasso method and glmnet

In this section we describe how we estimated the coefficients in β using the lasso (the least absolute shrinkage and selection operator) method ([11]). More specifically, we estimated the coefficients under the lasso penalty by minimizing the negative penalized log-likelihood in Eq. (7), that is,

$$\hat{\beta}(\lambda) = \text{argmin}_{\beta} \left(-\ell(\beta) + \lambda \sum_{j} |\beta_j| \right).$$

The estimation was done via a coordinate descent algorithm using the glmnet package ([5]) in the statistical package R. We chose the binomial family with logit link function in the settings of the glmnet function. Furthermore, the choice of λ was done by generating a sequence of 100 λ values and then choosing the best, using 10-fold cross-validation. All of those functions are provided by the package.

The glmnet function for the binomial model requires a two-level response. In our case we have, however, a constant response equal to one. To overcome this problem and allow the use the glmnet function, we added N rows of zeros to the data matrix X, and N new observations to the response vector Y, all with the value zero. We demonstrate below that this does not change the likelihood function, and since the response vector now contains both zeros and ones, the estimation of β can be done using the glmnet function,

To see why the above claim is true, let j correspond to lines of the extended data matrix X which were added, while i corresponds to lines of the original data matrix. Then $p_j = 1/(1+e^0) = 1/2$, while $p_i = \frac{1}{1+e^{-X_i\beta}}$, as before. Hence the likelihood in Eq. (6), using the extended data matrix, becomes

$$L(\beta) = \prod_{j=1}^{N} p_j^{y_j}(1-p_j)^{1-y_j} \prod_{i=1}^{N} p_i^{y_i}(1-p_i)^{1-y_i} = \prod_{j=1}^{N}(1-p_j)\prod_{i=1}^{N} p_i = (1/2)^N \prod_{i=1}^{N} p_i, \tag{8}$$

which equals the original likelihood in Eq. (6) multiplied by a constant independent of the parameters. Hence, the estimated coefficients are not affected by the modification. We chose N additional observations with zero response because, as we will discuss in Appendix A.4, we use bootstrapping to sample from the data matrix X. We found that N extra observations are enough to ensure both 0 and 1 responses in each bootstrap sample. If we alternatively added, say, only one extra observation, we would most probably get some bootstrap samples with only 1 as a response, and thus the glmnet function would not work. However, as already noted, the actual number of extra observations with zero response does generally not affect the estimated coefficients.

A.4 Bolasso

Lasso sometimes allows a few irrelevant variables to enter the model ([3]). Further, according to Tibshirani [11], the standard errors for the lasso estimates

are difficult to acquire. Those problems can be reduced by bootstrapping. Tibshirani [11], Avalos et al. [3] and Avalos et al. [2] suggested a bootstrap version of lasso which is called bolasso, which reduces the uncertainties of the lasso estimates. Bolasso runs a bootstrap on many samples, and estimates the parameters for each sample, using the lasso approach.

In our analysis we ran a bootstrap on the lasso model described in Section A.3, using 1000 bootstrap samples. For each bootstrap sample, a new λ sequence was generated by the glmnet package and the optimal λ was chosen using 10-fold cross validation. Then the model was fitted by glmnet using the current bootstrap sample.

The β-coefficients frequently selected by the lasso from the bootstrap samples are taken into account, while the others are set to zero [3]. Avalos et al. [3] suggested using Akaike's information criterion ([1]), $AIC = -2\ell(\hat{\beta}) + 2k$, for estimating the frequency threshold of bolasso. Here $\ell(\hat{\beta})$ is the log-likelihood function applied on the estimated coefficients from each model under selection, and k is the total number of non-zero elements in the β vector. AIC corresponds to an estimation of the allowed number of times that a coefficient could have been set to zero among the bootstraps. We can compute the AIC for different models and then choose the model which gives the lowest AIC-value.

The output from the bolasso in our analysis is a matrix of dimension $p \times B$. Each column of the matrix corresponds to one bootstrap replicate, that is, the estimated vector of coefficients from that bootstrap sample. Each row of the matrix corresponds to the values that the specific coefficient took among the bootstrap samples. For each row in the matrix, we computed the total number of zeros and we created a frequency vector of length p. For each value in the frequency vector we created a model by checking which of the coefficients had been set to zero less times than the chosen threshold. For those, their mean among the bootstraps was taken as an estimate, that is

$$\hat{\beta}_j = \sum_{b=1}^{B} \beta_j^{*b}/B,$$

where β_j^{*b} is the b-th bootstrap estimate of coefficient β_j and B is the total number of bootstrap replicates ([4]). The other coefficients are simply set to zero. Next, the AIC was computed for the corresponding model, where k is the number of non-zero estimates in the newly computed β vector. Finally, this is done for all thresholds in the sequence and the one which gives the lowest AIC is chosen as optimal. For that optimal threshold we again check which of the coefficients have been set to zero less times than the optimal threshold and we compute $\hat{\beta}_j$ as before, while the others are set to zero. This vector of coefficients is the bolasso estimate of the coefficients for the objective function ([3]).

Finally, we can compute the standard error of each coefficient as

$$S\hat{D}_j = \left(\sum_{b=1}^{B} \left(\beta_j^{*b} - \hat{\beta}_j \right)^2 / (B-1) \right)^{1/2},$$

as well as 95% confidence intervals based on the central limit theorem $(\hat{\beta}_j - 1.96S\hat{D}_j, \hat{\beta}_j + 1.96S\hat{D}_j)$ ([4]). However, according to Lockhart et al. [7], con-

fidence intervals obtained by the bootstrap estimates are not optimal for the lasso.

References

[1] Hirotogu Akaike. *Selected Papers of Hirotugu Akaike*, chapter Information Theory and an Extension of the Maximum Likelihood Principle, pages 199–213. Springer New York, New York, NY, 1998.

[2] Marta Avalos, Yves Grandvalet, Nuria Duran Adroher, Ludivine Orriols, and Emmanuel Lagarde. Analysis of multiple exposures in the case-crossover design via sparse conditional likelihood. *Stat Med*, 2012.

[3] Marta Avalos, Ludivine Orriols, Helene Pouyes, Yves Grandvalet, Frantz Thiessard, and Emmanuel Lagarde. Variable selection on large case-crossover data: application to a registry-based study of prescription drugs and road traffic crashes. *Pharmacoepidemiol Drug Saf*, 2013.

[4] B. Efron and R.J. Tibshirani. *An Introduction to the Bootstrap*. Chapman & Hall/CRC Monographs on Statistics & Applied Probability. Taylor & Francis, 1994.

[5] Jerome H. Friedman, Trevor Hastie, and Rob Tibshirani. Regularization paths for generalized linear models via coordinate descent. *Journal of Statistical Software*, 33(1):1–22, 2 2010.

[6] Holly Janes, Lianne Sheppard, and Thomas Lumley. Overlap bias in the case-crossover design, with application to air pollution exposures. *Statistics in Medicine*, 24(2):285–300, 2005.

[7] Richard Lockhart, Jonathan Taylor, Ryan J. Tibshirani, and Robert Tibshirani. A significance test for the lasso. 2013.

[8] Thomas Lumley and Drew Levy. Bias in the case – crossover design: implications for studies of air pollution. *Environmetrics*, 11(6):689–704, 2000.

[9] J.M. Marin and C. Robert. *Bayesian Core: A Practical Approach to Computational Bayesian Statistics*. Springer Texts in Statistics. Springer, 2007.

[10] G. Rodríguez. Lecture notes on generalized linear models. 2007.

[11] Robert Tibshirani. Regression shrinkage and selection via the lasso. *Journal of the Royal Statistical Society, Series B*, 58:267–288, 1994.
